# Cold Acclimation Favors Metabolic Stability in *Drosophila suzukii*

**DOI:** 10.3389/fphys.2018.01506

**Published:** 2018-11-01

**Authors:** Thomas Enriquez, David Renault, Maryvonne Charrier, Hervé Colinet

**Affiliations:** ^1^ECOBIO – UMR 6553, Université de Rennes 1, CNRS, Rennes, France; ^2^Institut Universitaire de France, Paris, France

**Keywords:** spotted wing drosophila, cold tolerance, cold shock, homeostasis, recovery, metabolites, metabotype

## Abstract

The invasive fruit fly pest, *Drosophila suzukii*, is a chill susceptible species, yet it is capable of overwintering in rather cold climates, such as North America and North Europe, probably thanks to a high cold tolerance plasticity. Little is known about the mechanisms underlying cold tolerance acquisition in *D. suzukii*. In this study, we compared the effect of different forms of cold acclimation (at juvenile or at adult stage) on subsequent cold tolerance. Combining developmental and adult cold acclimation resulted in a particularly high expression of cold tolerance. As found in other species, we expected that cold-acclimated flies would accumulate cryoprotectants and would be able to maintain metabolic homeostasis following cold stress. We used quantitative target GC-MS profiling to explore metabolic changes in four different phenotypes: control, cold acclimated during development or at adult stage or during both phases. We also performed a time-series GC-MS analysis to monitor metabolic homeostasis status during stress and recovery. The different thermal treatments resulted in highly distinct metabolic phenotypes. Flies submitted to both developmental and adult acclimation were characterized by accumulation of cryoprotectants (carbohydrates and amino acids), although concentrations changes remained of low magnitude. After cold shock, non-acclimated chill-susceptible phenotype displayed a symptomatic loss of metabolic homeostasis, correlated with erratic changes in the amino acids pool. On the other hand, the most cold-tolerant phenotype was able to maintain metabolic homeostasis after cold stress. These results indicate that cold tolerance acquisition of *D. suzukii* depends on physiological strategies similar to other drosophilids: moderate changes in cryoprotective substances and metabolic robustness. In addition, the results add to the body of evidence supporting that mechanisms underlying the different forms of acclimation are distinct.

## Introduction

Extreme temperatures often negatively affect survival of ectothermic animals as well as their biological functions such as reproduction, respiration, digestion, or excretion (Chown and Nicolson, 2004; Angilletta, 2009). In order to reduce the negative effects of temperature on their performances, ectotherms are capable of modulating thermal tolerance during their lifetime using a range of physiological adjustments that take place after pre-exposure to sub-lethal temperatures, a phenomenon referred to as thermal acclimation (Angilletta, 2009; Colinet and Hoffmann, 2012). The degree of tolerance acquisition directly depends on the thermal history of individuals, more particularly on the timing and length of the pre-exposure (Chown and Nicolson, 2004). The capacity to plastically deal with thermal stress is also believed to be a key factor in the success of exotic invasive species (Davidson et al., 2011; Renault et al., 2018).

Developmental plasticity can irreversibly alter some phenotypic traits, such as morphology (Piersma and Drent, 2003). For example, insects developing at low temperature are characterized by a larger body size and darker cuticle pigmentation of adults that remains throughout their whole life (Gibert et al., 2000, 2007). However, physiological adjustments occurring during development, like those related to acquired cold tolerance, are not necessarily everlasting (Piersma and Drent, 2003). For instance, cold tolerance acquired during development is readily adjusted to the prevailing conditions during adult acclimation without a detectable developmental constraint (Slotsbo et al., 2016). The different forms of acclimation probably lie along a continuum of shared common mechanisms; however, several lines of evidence suggest that physiological underpinnings of each acclimation form show some specificity (Colinet and Hoffmann, 2012; Teets and Denlinger, 2013; Gerken et al., 2015).

Stressful low temperatures compromise cells’ integrity by altering cytoskeleton structures and membranes’ functions (Cottam et al., 2006; Lee et al., 2006; Denlinger and Lee, 2010; Des Marteaux et al., 2017). Cold stress also induces central nervous system shutdown and loss of ions and water homeostasis that result in coma and neuromuscular impairments (Koštál et al., 2004; MacMillan and Sinclair, 2011; Andersen et al., 2016). Alteration of metabolic homeostasis is another symptom of cold stress, likely resulting from downstream consequences such as loss of function of membranes and enzymes (Overgaard et al., 2007; Teets et al., 2012; Williams et al., 2014, 2016; Colinet et al., 2016; Koštál et al., 2016b; Colinet and Renault, 2018). Thermal acclimation likely depends on many concomitant physiological adjustments such as changes in membrane fluidity (e.g., Overgaard et al., 2005; Lee et al., 2006; Koštál et al., 2011a; Williams et al., 2014), preservation of membrane potential and ion balance (Andersen et al., 2016; Overgaard and MacMillan, 2016), maintenance of metabolic homeostasis (e.g., Malmendal et al., 2006; Colinet et al., 2012; Teets et al., 2012), altered expression of heat shock proteins (Colinet and Hoffmann, 2012), and accumulation of substances with cryoprotective functions, such as sugars, polyols and amino acids (Koštál et al., 2011a; Vesala et al., 2012; Foray et al., 2013; MacMillan et al., 2016). Cryoprotective solutes can have beneficial effects at high concentration, by decreasing hemolymph freezing temperature (colligative effect) (Zachariassen, 1985; Storey and Storey, 1991), but also at low concentration, by stabilizing membranes and protein structures (Carpenter and Crowe, 1988; Crowe et al., 1988; Yancey, 2005; Cacela and Hincha, 2006).

The spotted wing drosophila, *Drosophila suzukii*, is an invasive species that is now spread in West and East Europe (Calabria et al., 2012; Lavrinienko et al., 2017) as well as in North and South America (Hauser, 2011; Lavagnino et al., 2018; see also Asplen et al., 2015 for a review). Contrary to other drosophilids, *D. suzukii* females lay eggs in mature fruits. Larvae consume these fruits, causing important damages and economic losses to a wide range of fruit crops (Goodhue et al., 2011; Walsh et al., 2011). This fly is highly polyphagous and is therefore able to develop on a wide range of wild fruits in addition to those that are cultivated (Poyet et al., 2015; Kenis et al., 2016). This fly is chill susceptible, succumbing to temperatures well above 0°C (Kimura, 2004; Dalton et al., 2011; Ryan et al., 2016; Enriquez and Colinet, 2017). Yet, *D. suzukii* is capable of overwintering in rather cold climates, such as in Northern America and Europe, probably by using various strategies such as migration to favorable microhabitats (Zerulla et al., 2015; Rossi-Stacconi et al., 2016; Tonina et al., 2016; Thistlewood et al., 2018) and/or high cold tolerance plasticity. These strategies allow maintenance of the populations in invaded areas, even if the number of adults is drastically reduced during winter (Mazzetto et al., 2015; Arnó et al., 2016; Wang et al., 2016).

*Drosophila suzukii* is capable of enhancing cold tolerance via a range of acclimation responses (Hamby et al., 2016). Jakobs et al. (2015) found that exposure at 0°C for 1 h did not trigger a rapid cold hardening response in *D. suzukii*. Conversely, certain lines or populations of *D. suzukii* actually exhibit a typical rapid cold hardening response. Toxopeus et al. (2016) showed that flies acclimated during development could show a rapid cold hardening response after 1 or 2 h at 0°C. Everman et al. (2018) found a similar rapid cold-hardening response in non-acclimated flies exposed at 4°C for 2 h. This fly is also capable of acquiring cold tolerance via acclimation at adult stage (Jakobs et al., 2015; Wallingford and Loeb, 2016), or via developmental acclimation (Toxopeus et al., 2016; Wallingford and Loeb, 2016). In *D. suzukii*, developmental acclimation at temperatures below 12°C (combined or not with short photoperiod) results in a phenotype showing increased body size, dark pigmentation, reproductive arrest, and enhanced cold tolerance; this phenotype, referred to as “winter morph”, is supposed to be the overwintering form of *D. suzukii* (Stephens et al., 2015; Shearer et al., 2016; Toxopeus et al., 2016; Wallingford and Loeb, 2016; Everman et al., 2018). Effect of the different forms of acclimation on subsequent cold tolerance has been rather well described in *D. suzukii*, but surprisingly, the mechanisms underlying cold tolerance acquisition through acclimation in this species are still poorly understood. In order to better appreciate and predict overwintering strategies of *D. suzukii*, knowledge about its thermal biology, and particularly cold stress physiology, is urgently needed (Asplen et al., 2015; Hamby et al., 2016).

A recent transcriptomic study suggested that cold tolerance of winter morphs is associated with an upregulation of genes involved in carbohydrates’ metabolism (Shearer et al., 2016). However, it is still not known whether cold-hardy *D. suzukii* flies use any specific cryoprotective arsenal. In this study we proposed the first characterization of metabolic adaptations linked to cold acclimation in *D. suzukii*. First, we aimed at assessing the impact of different forms of acclimation on cold tolerance in this species. To do so, we subjected flies to developmental acclimation, adult acclimation or a combination of both acclimation forms, and then assessed subsequent cold tolerance of adults. We expected that (i) each cold acclimation form would promote cold tolerance, and that (ii) combining cold acclimation during both development and adult stage would further promote cold tolerance. Second, we intended to explore underlying mechanisms of cold acclimation in *D. suzukii*. Specifically, we performed quantitative target gas chromatography–mass spectrometry (GC-MS) profiling to explore metabolic changes, including accumulation of cryoprotectants, in four different phenotypes resulting from different thermal treatments. We also performed a time-series GC-MS analysis to monitor metabolic homeostasis status during cold stress and recovery. We expected that, as other *Drosophila* species (Overgaard et al., 2007; Koštál et al., 2011a; Colinet et al., 2012; Vesala et al., 2012; MacMillan et al., 2016), cold acclimated *D. suzukii* would be characterized by increased concentrations of some cryoprotectants (sugars, polyols, or amino acids) and would be able to maintain metabolic homeostasis after cold shock contrary to chill susceptible phenotypes.

## Materials and Methods

### Flies Rearing and Thermal Treatments

In this work, we used a wild-type *D. suzukii* population coming from the Vigalzano station of the Edmund Mach Foundation (Italy; 46.042574N, 11.135245E). This line was established in 2011 in Italy, and was sent to our laboratory (Rennes, France) in 2016. For the experimentations, *D. suzukii* was reared in glass bottle (100 mL) supplied with a carrot-based food (agar: 15 g, sucrose: 50 g, carrot powder: 50 g, brewer yeast: 30 g, cornmeal: 20 g, Nipagin: 8 mL, tap water: 1 L). Flies were maintained at 25°C, 60% RH, 12 L:12 D into incubators (Model MIR-154-PE; PANASONIC, Healthcare Co., Ltd., Gunma, Japan). At least 12 bottles (each containing 100–300 flies) were used to continuously maintain the line at Rennes, and flies from different bottles were crossed every generation to limit inbreeding.

**FIGURE 1:**
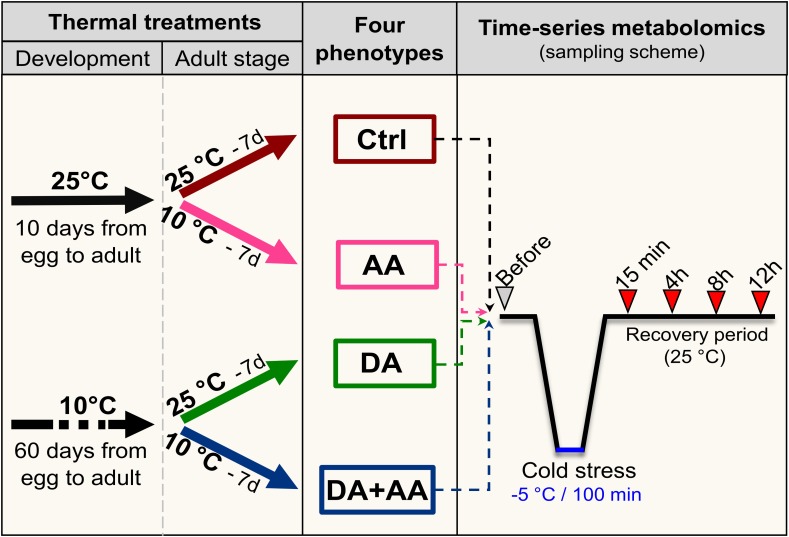
Thermal treatments experienced by flies during development (25°C or developmental acclimation [DA] at 10°C), and at adult stage (25°C or adult acclimation [AA] at 10°C), leading to the four treatments groups, and sampling scheme used for the time-series metabolic analysis. Ctrl, control flies; DA, developmental acclimation; AA, adult acclimation; DA + AA, combined developmental and adult acclimation.

To generate flies for the experiments, groups of approximately 100 mature (7 days old) males and females were placed in 100 mL bottles containing food medium, and females were allowed to lay eggs during 48 h at 25°C. Flies were then removed and bottles with eggs were randomly placed under the different thermal treatments to continue development (Figure 1). Bottles containing eggs were directly placed either at 25°C (12 L:12 D) [no acclimation] or cold acclimated at 10°C (10 L:14 D) [developmental acclimation] (Figure 1). Flies took about 10 and 60 days to start emergence at 25 and 10°C, respectively. At emergence, adults from both developmental conditions were directly placed either at 25°C (12 L:12 D) [control, no acclimation] or cold acclimated at 10°C (10 L:14 D) [adult acclimation] with fresh food for seven consecutive days. Flies were transferred into new bottles containing fresh food every 2 days. Thereby, four different phenotypes that experienced four different thermal treatments were generated: non-acclimated control, adult acclimation, developmental acclimation and combined developmental and adult acclimation hereafter referred to as combined acclimation (Figure 1). All experiments were conducted on males, which were separated from females visually (with an aspirator) without CO_2_ to avoid stress due to anesthesia (Colinet and Renault, 2012).

### Recovery From Acute Cold Stress

Males randomly taken from each treatment group were distributed in 10 replicates of 10 individuals and were subjected to an acute cold stress using 10 glass vials that were directly immersed in a glycol solution cooled by a cryostat (Cryostat Lauda ECO RE 630) at -5°C for 100 min. In a previous experiment, this combination of temperature and duration induced 40% survival in non-acclimated *D. suzukii* (Enriquez and Colinet, 2017). After exposure, flies were directly transferred in 40 mL food vials and allowed to recover at 25°C (12 L: 12 D). Survival was assessed by counting the number of dead and living individuals in each vial 4, 24, and 48 h after the acute cold stress.

### Critical Thermal Minimum (Ct_min_)

To measure Ct_min_, we used a glass knockdown column that consisted of a vertical jacketed glass column (52 × 4.7 cm) containing several cleats to help flies not fall out the column while still awake. In order to regulate the temperature, the column was connected to a cryostat (Cryostat Lauda ECO RE 630), and temperature was checked into the column using a thermocouple K connected to a Comark Tempscan C8600 scanning thermometer (Comark Instruments, Norwich, United Kingdom). Thermocouple was positioned at the column center, at mid height through a tiny insulated hole. For each condition, approximately 60 flies were introduced at the top of the column. Flies were allowed to equilibrate in the device for few minutes, and then the temperature was decreased to -5°C at 0.5°C/min. For each individual fly falling out of the column, the Ct_min_ (°C) was recorded.

### Chill Coma Recovery Time

Chill coma recovery time (CCRT) is defined as the resurgence time of motor activity after a cold knockdown (David et al., 1998). In order to induce coma, 40 males of each experimental condition were subjected to 0°C for 12 h, using a vial placed directly in a cooled-incubator. We choose this temperature and duration because in a previous experiment we showed that 12 h at 0°C induced a relatively small mortality level in non-acclimated males (Enriquez and Colinet, 2017). Upon removal, adults were positioned supinely on a table in a thermally controlled room (25 ± 1°C), using a fine paintbrush, and the time to regain the ability to stand (i.e., CCRT) was monitored individually. Experimentation lasted 60 min, and flies that did not recover by that time were marked as not recovered (censored).

### GC-MS Metabolic Profiling

To assess the effect of the different thermal treatments on metabolic profiles, we sampled flies at the end of each thermal treatment period (i.e., before the stress exposure) (Figure 1). Then, males randomly taken from each treatment group were cold-stressed at -5°C for 100 min (as explained above), and these stressed flies were then sampled during the recovery period after 15 min, 4, 8, and 12 h (Figure 1). For each time-point, seven replicates, each consisting of a pool of 10–12 living flies, were snap-frozen in liquid N_2_ and stored at -80°C. Fresh mass of each sample was measured prior to metabolites’ extraction using a microbalance (Mettler Toledo UMX2, accuracy 0.001 mg).

Samples were homogenized using a bead-beating device (Retsch MM301, Retsch GbmH, Haan, Germany) at 20 beats per second during 90 s in 600 μL of a cold solution of methanol-chloroform (2:1) with a tungsten grinding ball. Samples were then kept at -20°C for 30 min. A volume of 400 μL of ultrapure water was added to each tube, and samples were vortexed. After centrifugation at 4000 *g* for 10 min at 4°C, 120 μL aliquots of the upper aqueous phase containing polar metabolites were transferred to microtubes and vacuum-dried (SpeedVac Concentrator, miVac, Genevac Ltd., Ipswich, England). The dried polar phase aliquots were resuspended in 30 μL of 20 mg mL^-1^ methoxyamine hydrochloride (Sigma-Aldrich, St. Louis, MO, United States) in pyridine, and incubated under orbital shaking at 40°C for 60 min. Afterward, 30 μL of N,O-Bis(trimethylsilyl)trifluoroacetamide (BSTFA; Sigma-Aldrich) was added, and derivatization was performed at 40°C for 60 min under continuous agitation.

Gas chromatography/mass spectrometry was used to quantify primary metabolites (non-structural carbohydrates, polyols, amino and organic acids), as described in Colinet et al. (2016). Briefly, GC-MS consisted of a CTC CombiPal autosampler (CTC Analytics AG, Zwingen, Switzerland), a Trace GC Ultra chromatograph, and a Trace DSQII quadrupole mass spectrometer (Thermo Fischer Scientific Inc., Waltham, MA, United States). The autosampler enabled online derivatization and standardization of the preparation process: each sample was automatically prepared during GC analysis of the preceding sample, ensuring the highest possible throughput of the system, and resulting in equal derivatization duration for each compound prior to injection. For each sample, 1 μL was injected in the oven using the split mode (25:1; temperature of the injector: 250°C). The oven temperature ranged from 70 to 147°C at 9°C min^-1^, from 147 to 158°C at 0.5°C min^-1^, from 158 to 310°C at 5°C min^-1^ and held at 320°C for 4 min. Helium was the gas carrier (1 mL min^-1^) and MS detection was achieved using electron impact. All samples were run under the SIM mode (electron energy: -70 eV), thereby, we only screened for the 59 pure reference compounds included in our spectral database. GC-MS peaks were accurately annotated using both mass spectra (two specific ions), and retention index specific to each compound. Calibration curve samples for 59 pure reference compounds at 10, 20, 50, 100, 200, 500, 700, and 1000 μM concentrations were run. Chromatograms were deconvoluted using XCalibur 2.0.7, and metabolite levels were quantified using the quadratic calibration curves for each reference compound and concentration. Concentrations were then normalized with the sample weight.

### Statistical Analyses

All analyses (except survival analyses of CCRT curves) were conducted using R (version 3.4.3; R Core Team, 2016). We modeled survival of flies exposed at -5°C by specifying a generalized linear mixed-effects model (GLMM) with logit link function for proportions outcome (i.e., number of dead/alive flies per vial). The response variable was dependent on the developmental temperature (10 vs. 25°C), the temperature at adult stage (10 vs. 25°C), the time to survival measurement and the interaction among terms. Vial number was considered as random effect. We analyzed the effect of each variable through an analysis of deviance (“Anova” function in “car” package, Fox and Weisberg, 2011). Differences among treatment groups were analyzed by comparing least-squares means using the “emmeans” package (Russell, 2018).

Ct_min_ data were analyzed using a two-way ANOVA, dependent on developmental (10 vs. 25°C) and adult temperatures (10 vs. 25°C), and the interaction between these two factors. Then, a Tukey HSD comparison was used to identify differences between the interaction of these two factors.

CCRT data were analyzed using survival analyses with the software GraphPad Prism5. We compared recovery curves using Gehan-Breslow-Wilcoxon Tests. We adjusted the alpha level of significance thanks to Bonferroni correction (α = 0.008).

Concentrations of each metabolite after the four thermal treatments have been checked for normality: for some metabolites, normality was not respected, but total number of individuals and sample size were sufficient to use one-way ANOVAs (Blanca et al., 2017). Tukey HSD comparisons were then used to identify differences between thermal treatments. Metabolite concentrations were also pooled in groups corresponding to their respective biochemical families (i.e., amino acids, carbohydrates, polyols, etc.). Similarly, concentrations of biochemical families were analyzed using one-way ANOVAs followed by Tukey tests.

If, as speculated, cold acclimated phenotypes are characterized by a post-stress metabolic inertia, while non-acclimated counterparts loose metabolic homeostasis, then, different temporal patterns should be observed during recovery period among phenotypic groups. This should result in significant time × treatment interactions, at both univariate and multivariate levels. Temporal changes in the concentrations of metabolites and biochemical families were analyzed using GLMs with Gaussian link function, and the effect of the interaction between the thermal treatments and time after the cold stress were analyzed through an analysis of deviance (“Anova” function in “car” package, Fox and Weisberg, 2011).

Data resulting from quantitative GC-MS analysis were also analyzed using several multivariate tests. The metabolites’ compositions resulting from each phenotype (i.e., the metabotype) were compared using between-class principal component analysis (PCA) (Dolédec and Chessel, 1991) in the “ade4” package in R (Dray and Dufour, 2007). A first PCA was performed using metabolic datasets of the four treatments before stress exposure (see Figure 1), in order to identify main patterns and clustering resulting from the different thermal conditions. A second PCA was performed on the time-series datasets of the four phenotypes in order to monitor metabolic homeostasis status during recovery period (see Figure 1). Monte Carlo tests were performed to examine the significance of the difference among the classes (based on 1000 simulations). To identify the variables (i.e., metabolites) contributing the most to the PCA structure separation, the correlations to the principal components (PCs) were extracted and ranked. Data were scaled and mean-centered prior to the PCAs. In addition to the classical PCA, an ASCA was performed to further analyze the time-series datasets. The ASCA (ANOVA-Simultaneous Component Analysis) is a multivariate approach ideal for the analysis of time-series metabolomic studies (Smilde et al., 2005). ASCA applies a PCA to the estimated parameters for each source of variation of the model and tests the main effects (i.e., thermal treatment and time) as well as the interaction. If acclimated phenotypes have a distinct and more efficient homeostatic response than control chill-susceptible group, then different temporal metabolic trajectories should be observed, and this should result in a significant interaction effect in the ASCA. The ASCA was performed using the temporal analysis module of MetaboAnalyst 4.0 (Chong et al., 2018). This pipeline tests the main and the interaction effects via permutation tests.

## Results

### Cold Tolerance

#### Survival to Acute Cold Stress

**FIGURE 2:**
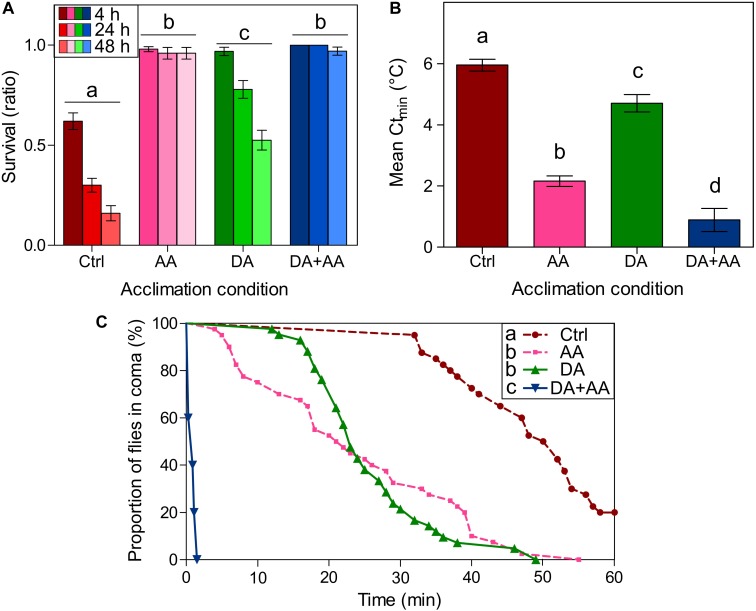
*Drosophila suzukii* cold survival according to thermal treatments. Flies have been subjected to an acute cold stress at -5°C for 100 min **(A)**. Survival was assayed 4, 24, and 48 h after cold shock. Bars represents survival probability ± standard error of the mean (SEM). Phenotypes with the same letters are not significantly different, disregarding the temporal effect (*p*-value < 0.05; least-squares means comparisons); *n* = 100 flies per condition. *D. suzukii* critical thermal minimum (Ct_min_) depending on thermal treatments **(B)**. Bars represents mean Ct_min_ ± Standard Error of the Mean (SEM). Groups with the same letters are not significantly different (*p*-value < 0.05; Tukey HSD); *n* = minimum 60 flies per condition. Chill coma recovery time (CCRT) according to thermal treatments **(C)**. Flies were submitted to 0°C for 12 h and then CCRT was recorded at 25°C. Each point corresponds to the recovery time of one fly. Recovery curves were analyzed using survival analyses. Groups with the same letters are not significantly different (*p*-value < 0.008; Gehan-Breslow-Wilcoxon tests); *n* = 40 flies per condition; Ctrl, control flies; DA, developmental acclimation; AA, adult acclimation; DA + AA, combined developmental and adult acclimation.

Figure 2A illustrates survival probability of the flies 4, 24, and 48 h after a cold shock (-5°C) in the four phenotypes. Flies from combined acclimation were the only showing 100% survival, at 4 and 24 h post stress. GLMM model showed an effect of developmental conditions and rearing temperature at adult stage on cold survival (χ^2^ = 40.41, *df* = 1; *p*-value < 0.001; χ^2^ = 107.36, *df* = 1, *p*-value < 0.001, respectively). Survival decreased with increased time of assessment after cold exposure, particularly in control and developmental acclimation (χ^2^ = 84.98, df = 1, *p*-value < 0.001). Outcomes from least-square means comparisons are available on Figure 2A: globally, flies from combined acclimation and adult acclimation showed the highest survival rates (97 and 96% after 48 h, respectively), followed by flies submitted to developmental acclimation and control group that showed the lowest survival rate (52 and 16% after 48 h, respectively).

#### Ct_min_

Ct_min_ values of the four phenotypes are illustrated in Figure 2B. Ct_min_ was affected by developmental conditions and temperature at adult stage [*F*_(1,215)_ = 26.56, *p*-value < 0.001; *F*_(1,215)_ = 230.20, *p*-value < 0.001, respectively]. Outcomes from Tukey HSD comparisons are available on Figure 2B: Ct_min_ values of the four phenotypes were all significantly different, flies from combined acclimation had the lowest Ct_min_ (0.89°C), followed by flies from adult acclimation (2.16°C), developmental acclimation (4.70°C), and finally control flies which had the highest Ct_min_ (5.95°C).

#### CCRT

CCRT curves are displayed in Figure 2C. In flies from combined acclimation group, it took no more than two minutes for the flies to recover, in the other groups recovery took much longer. Consequently, flies from combined acclimation were the very first to recover, followed by flies submitted to adult acclimation and developmental acclimation, and finally controls flies took the longer time to recover: after one hour of recovery, 20% of the control flies were still in coma. Outcomes from the Gehan-Breslow-Wilcoxon tests are available on Figure 2C.

### Metabolic Characterization of Acclimation

#### Metabolite Concentrations After Acclimation

**FIGURE 3:**
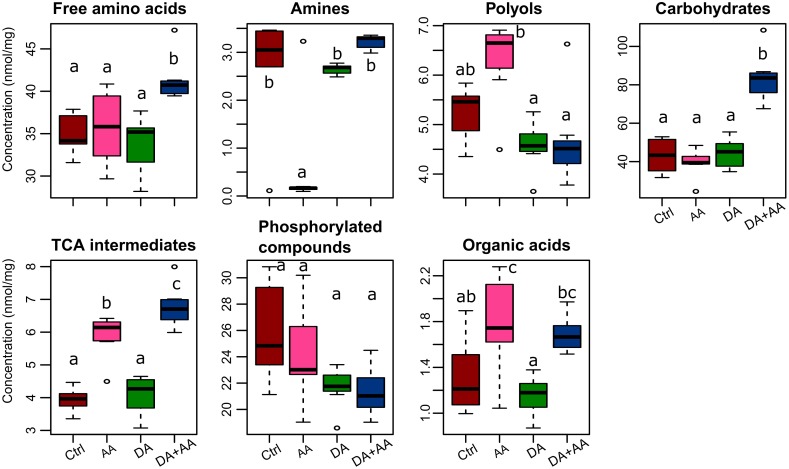
Concentrations of the different biochemical families after the different thermal treatments. Boxplot sharing a same letter are not significantly different (*p*-value < 0.05; Tukey test); *n* = approximately 80 flies per condition. Ctrl, control flies; DA, developmental acclimation; AA, adult acclimation; DA + AA, combined developmental and adult acclimation.

Among the 59 metabolites included in our spectral library, 45 were identified and quantified (i.e., absolute quantification). The list of identified metabolites, their respective abbreviations and their biochemical families are presented in Supplementary Table S1. Figure 3 presents the variation patterns by biochemical classes at the end of thermal treatments. Outcomes resulting from Tukey tests on the different biochemical families according to thermal treatments are available on Figure 3. Except for phosphorylated compounds, mean concentrations of all biochemical families varied according to thermal conditions experienced during development and as adult (Figure 3). The most striking changes were observed in flies submitted to combined acclimation that were characterized by significant increases in free amino acids, carbohydrates and tricarboxylic acid (TCA) intermediates in comparison to control flies (1.15-, 2-, and 6-fold change, respectively; Figure 3). Flies from adult acclimation displayed a significant decrease in amines and increased levels of TCA intermediates relative to control (0.03- and 1.5-fold change, respectively; Figure 3). These global patterns were driven by concentration changes in individual’s metabolites that are all available in the panel Supplementary Figures S1–S7. Flies from the combined acclimation showed high amounts of several free amino acids (Ala, Glu, Ile, Leu, Phe, Ser, Val; Supplementary Figure S1), carbohydrates (Fru, Mal, Man, Suc, Tre; Supplementary Figure S4), TCA intermediates (citrate, malate, succinate; Supplementary Figure S5), and the other compounds GABA and GDL (Supplementary Figure S7). In this phenotypic group, a decrease of F6P and G6P was observed compared to control (Supplementary Figure S6). In comparison to control group, flies submitted to adult acclimation were characterized by an accumulation of several polyols (glycerol, mannitol, and xylitol; Supplementary Figure S3) and two TCA intermediates (malate, citrate; Supplementary Figure S5) and a decrease in spermine, F6P and G6P (Supplementary Figures S2, S6). Patterns of flies submitted to developmental acclimation were relatively similar to those of control flies, only two amino acids had increased amounts (Ser and Thr), while three compounds had decreased concentrations (Ala, Cit, and Phe) (Supplementary Figure S1).

#### Multivariate Analysis on Metabotypes After Acclimation

**FIGURE 4:**
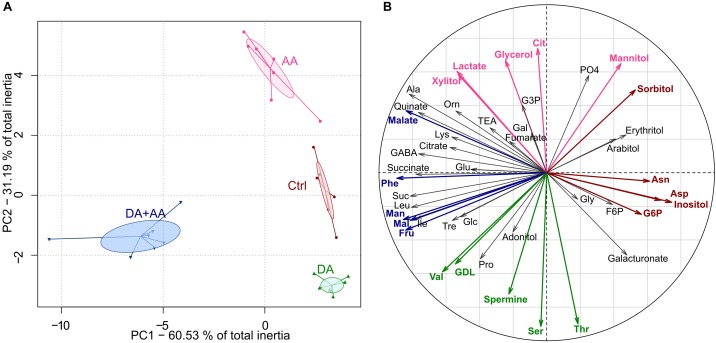
Principal component analyses (PC1 vs. PC2) on the 45 metabolite concentrations after the different thermal treatments; *n* = approximately 80 flies per condition **(A)**. Correlation circle of the 45 metabolites after acclimation **(B)**. Metabolites highlighted in red or fuchsia correspond to the five metabolites contributing the most positively to PC1 and PC2, respectively; Metabolites highlighted in blue or green correspond to the five metabolites contributing the most negatively to PC1 and PC2, respectively. Ctrl, control flies; DA, developmental acclimation; AA, adult acclimation; DA + AA, combined developmental and adult acclimation.

Global metabolic changes according to thermal treatments were also characterized using a PCA, and the ordination of classes within the first plane is presented in Figure 4A. This multivariate analysis revealed a clear-cut non-overlapping separation among the four thermal treatments (Figure 4A). The metabotype reflecting combined acclimation was the most divergent and was opposed to the other metabotypes on the first axis of the PCA (PC1, 60.53% of total inertia). The three other metabotypes separated along the second axis (PC2, 31.19% of total inertia). Thus, PC1 and PC2 supported 91.72% of total inertia. The Monte-Carlo randomizations corroborated the significance of the differences among classes (i.e., treatments; observed *p*-value < 0.001). The projection of the variables of the PCA (i.e., metabolites) on the correlation circle are shown in panel Figure 4B and mirror concentration changes of individual metabolites (Supplementary Figures S1–S7).

### Temporal Patterns of Metabolic Profiles During the Recovery From Acute Cold Stress

#### Variation of Metabotypes During Recovery From Acute Cold Stress

**FIGURE 5:**
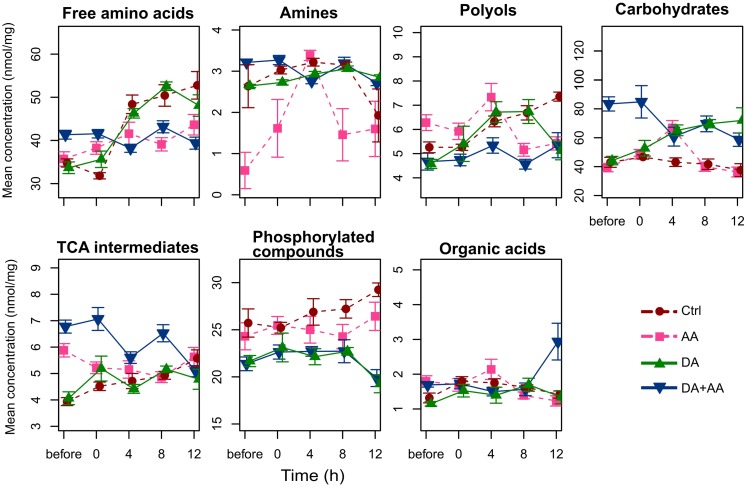
Concentration of the different metabolite categories during the recovery from a cold stress at -5°C for 100 min (before, 0, 4, 8, and 12 h after the cold stress); *n* = approximately 80 flies per condition per time point. Ctrl, control flies; DA, developmental acclimation; AA, adult acclimation; DA + AA, combined developmental and adult acclimation.

Metabolic compositions of the four thermal treatments were monitored before the stress, and during the post-stress recovery period. Figure 5 displays temporal changes of concentrations of metabolites’ biochemical families. Univariate statistics from GLMs describing the effects of recovery time, thermal treatments and their interaction are available in Table 1. Interactions (time × treatment) were found for all the biochemical families (Figure 5 and Table 1) suggesting divergent homeostatic trajectories among thermal treatments. This was reflected for instance in the pool of free amino acids or polyols that remained relatively stable in flies submitted to combined acclimation, whereas it increased in other phenotypes (Figure 5). Flies from combined acclimation were the only to show temporal decrease in global concentration of carbohydrates (0.75-fold change) and TCA intermediates dropped from 7 to 5 nmol/mg after 12 h of recovery (Figure 5). These global temporal patterns were driven by concentration changes in individual’s metabolites that are all available in the panel Supplementary Figures S8–S14, together with the statistics showing the significance of treatment x time interactions in Supplementary Table S2. Most of the metabolites showed significant treatment × time interaction (Supplementary Table S2). During the recovery from cold stress, control flies were characterized by increased concentrations in most metabolites, including free amino acids (Cit, Glu, Ile, Leu, Lys, Orn, Phe, Pro, Thr, and Val; Supplementary Figure S8), polyols (adonitol, erythritol, glycerol, sorbitol, and xylitol; Supplementary Figure S10), TEA, one TCA intermediate (citrate), PO4, galacturonate and GDL (Supplementary Figures S9, S12–S14). Flies from developmental acclimation also showed temporal increase in the concentrations of free amino acids (e.g., Ala, Glu, Ile, Leu, Phe, Pro, Thr, and Val; Supplementary Figure S8). Most of the quantified polyols accumulated at 4 or 8 h post-stress and returned to their initial concentrations after 12 h of recovery (Supplementary Figure S10). Furthermore, these flies showed a temporal accumulation of several carbohydrates (Fru, Man, and Tre; Supplementary Figure S11) and GDL (Supplementary Figure S14), and a light decrease in the level of phosphorylated compounds (F6P and G6P; Supplementary Figure S13). Flies from the adult acclimation showed temporal variations in the level of several free amino acids (decrease of Ala, increase of Asp, Ile, Leu, Pro, Ser, and Val; Supplementary Figure S8), increased amounts of amines (TEA and spermine; Supplementary Figure S9), succinate and a decrease of the concentration of malate (Supplementary Figure S12). The concentration of several polyols (adonitol, erythritol, mannitol, and xylitol; Supplementary Figure S10), carbohydrates (Fru, Glc, Man, and Tre; Supplementary Figure S11), organic acids (galacturonate, lactate, and quinate) and GABA and GDL (Supplementary Figure S14) showed a similar temporal pattern: the concentration increased 4 h after the cold stress and returned to initial concentration within 8 or 12 h after the stress. Finally, flies submitted to combined acclimation showed a decrease in Ala and Phe concentration during recovery from cold stress (Supplementary Figure S8). Other free amino acids (Gly, Ile, Leu, Ser, and Val; Supplementary Figure S8) showed increased levels during the recovery period but returned to initial concentrations after 12 h of recovery. The level of carbohydrates (Fru, Glc, Mal, Man, and Tre; Supplementary Figure S11) and TCA intermediates (succinate and malate; Supplementary Figure S12) moderately decreased with recovery time, while lactate accumulated after the cold stress (Supplementary Figure S12).

**Table 1:** Outcomes of GLMs on metabolites’ biochemical family concentrations during the recovery from the cold stress.

Biochemical families	Parameter	χ^2^	df	*p*-Value
Free amino acids	Treatment	18.19	3	<0.001***
	Time	117.59	4	<0.001***
	Treatment × Time	97.80	12	<0.001***
Amines	Treatment	45.75	3	<0.001***
	Time	16.18	4	<0.01**
	Treatment × Time	34.03	12	<0.001***
Polyols	Treatment	35.00	3	<0.001***
	Time	27.58	4	<0.001***
	Treatment × Time	51.52	12	<0.001***
Carbohydrates	Treatment	107.05	3	<0.001***
	Time	7.56	4	0.1
	Treatment × Time	65.17	12	<0.001***
TCA intermediates	Treatment	92.47	3	<0.001***
	Time	7.94	4	0.09
	Treatment × Time	64.71	12	<0.001***
Phosphorylated compounds	Treatment	82.74	3	<0.001***
	Time	2.24	4	0.69
	Treatment × Time	22.81	12	<0.05*
Organic acids	Treatment	14.51	3	<0.01**
	Time	4.17	4	0.38
	Treatment × Time	55.45	12	<0.001***


*Treatment, acclimation treatment; Time, recovery time.*

#### PCA on Metabolite Compositions During Recovery From the Acute Cold Stress

**FIGURE 6:**
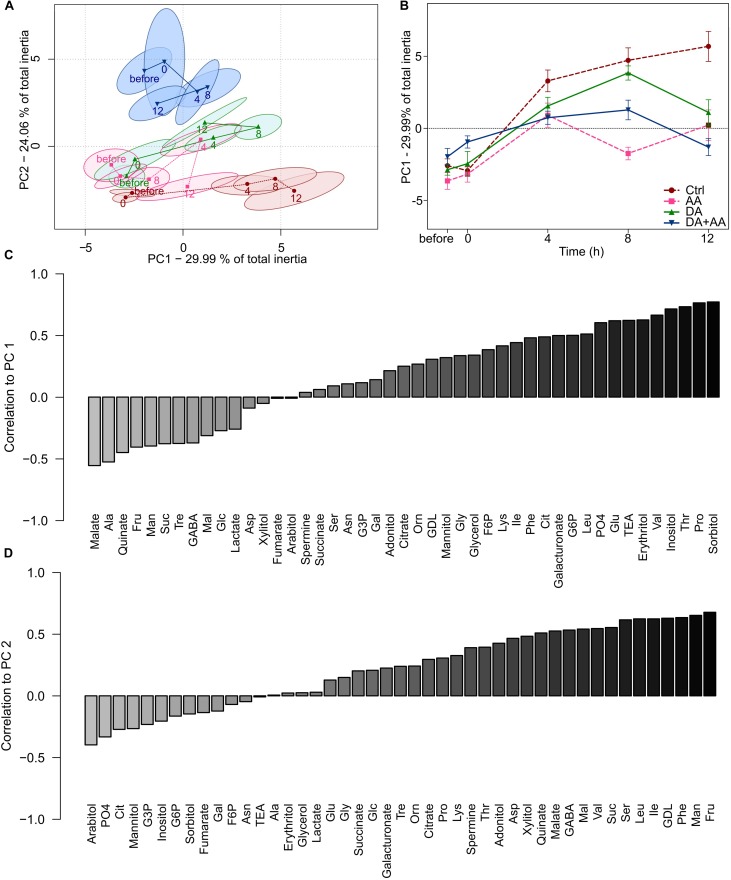
Principal component analyses (PC1 vs. PC2) on the 45 metabolite concentrations during the recovery from an acute cold stress at -5°C for 100 min (before, 0, 4, 8, and 12 h after the cold stress); *n* = approximately 80 flies per condition per time point **(A)**. Projection of the centroid scores of the PCA on PC1 according to recovery time (before, 0, 4, 8, and 12 h after the cold stress) **(B)**. Correlation values of the different concentrations of metabolites in relation to the principal components PC1 **(C)** and PC2 **(D)** in the PCA. Correlations are ranked on the Y-axis according to their values. Light and dark gray bars, respectively, correspond to metabolites negatively or positively correlated to PC-1 or -2. Ctrl: Control flies; DA: developmental acclimation; AA: adult acclimation; DA + AA: combined developmental and adult acclimation.

A PCA was made on metabolic patterns across the different sampling times (Figure 6A). Temporal changes among metabotypes were mainly reflected along PC1 (29.99% of total inertia), while differences between thermal treatments were associated with PC2 (24.06% of total inertia; PC1 + PC2 = 54.05% of total inertia). Metabotypes for control showed a marked temporal deviation along PC1. Metabotypes for adult and developmental acclimation also showed temporal deviations from initial status (especially after 8 h of recovery), but less pronounced than in the control group. Metabotypes for combined acclimation were again clearly distinct from the other groups on PC2 and showed a temporal deviation of metabolic status after 4 and 8 h of recovery, but after 12 h of recovery the metabolic profiles had returned to initial state on PC1 (Figure 6A). These differences among metabotypes were validated by Monte Carlo randomizations (observed *p*-value < 0.001). The temporal changes of these metabotypes can be observed in Figure 6B, which represents the projections of the centroid scores on PC1. From these, it can clearly be discerned that flies from control and developmental acclimation showed the most intense temporal deviations from initial states, while counterparts from combined acclimation showed much moderate deviation, and a returned to the initial metabolic state within 12 h. Flies from adult acclimation showed an intermediate response. Correlation of metabolites with PC1 and PC2 are available in Figures 6C,D, respectively. A large number of free amino acids (e.g., Pro, Thr, and Val) and polyols (e.g., erythritol, inositol, and sorbitol) were positively correlated to PC1 (Figure 6C), and these changes reflected temporal increase in metabolites’ concentrations, mainly in control flies (see Figure 5 and Supplementary Figure S2). Several carbohydrates (e.g., Fru, Man, Suc, and Mal) were positively correlated with PC2 (Figure 6D), revealing relative higher amounts of these metabolites in flies from combined acclimation compared to control flies (see also Figure 5 and Supplementary Figure S2).

#### ASCA on Metabolite Compositions During Recovery From Acute Cold Stress

The results of ASCA are presented in panel Supplementary Figure S15. The Supplementary Figure S15A shows the major patterns associated with the factor “treatment” (i.e., thermal treatments) of the ASCA. A decreasing trend was detected, with flies from combined acclimation showing positive score. Control flies were at the opposite, with a negative score and flies from developmental and adult acclimation were intermediate (Supplementary Figure S15A). This pattern mirrored the PCA results shown in Figure 6A. The Supplementary Figure S15B shows the major patterns associated with the factor “time”. The scores was initially negative (before the stress) and remained stable after the stress (0 h), then a marked increase occurred at 4 h of recovery (time 4), when the score became positive, after which the scores remained positive at 8 and 12 h of recovery. These results are also consistent with PCA results shown in Figures 6A,B, in which the metabotypes of flies showing the lowest cold tolerance (control and developmental acclimation mainly) shifted in the same direction on PC1 (toward positive scores) over the time-course of the experiment. Interestingly, the major patterns associated with interaction terms suggested that flies from combined and adult acclimation had rather similar temporal patterns that differed from that of flies from developmental acclimation and control (Supplementary Figure S15C), suggesting a major importance of adult acclimation. Finally, the observed statistics with permutation tests were significant for both main factors (observed *p*-value < 0.05 for both “treatment” and “time”), confirming the clear differentiation of metabotypes according to both “treatment” and “time” (Supplementary Figure S15D). More interestingly, the permutation test for “treatment × time” interaction was also significant (observed *p*-value < 0.05), demonstrating that temporal metabolic trajectories significantly differed according to thermal treatments (Supplementary Figure S15D).

## Discussion

### Impact of Thermal Acclimation on *D. suzukii* Cold Tolerance

In this study, we subjected *D. suzukii* flies to four different thermal treatments to estimate the effect of developmental and adult acclimation or the combination of these two forms of acclimation on cold tolerance of adults. As expected, combining developmental and adult acclimation led to the highest cold tolerance of flies, based on three different measures of cold tolerance (survival, Ct_min_, CCRT; Figure 2). These results confirm previous observations of Stephens et al. (2015), Shearer et al. (2016), or Toxopeus et al. (2016), who showed that combination of developmental and adult acclimation leads to a highly cold tolerant phenotype in *D. suzukii*. In *Drosophila melanogaster*, the combination of developmental and adult acclimation also resulted in cumulative effects (Colinet and Hoffmann, 2012; Slotsbo et al., 2016), but this pattern is not a general rule in insects (Terblanche and Chown, 2006). The fact that acclimation benefits cumulated in *D. suzukii* further adds to the widely accepted vision that physiological underpinnings of the different forms of acclimation are somewhat specific (Colinet and Hoffmann, 2012; Teets and Denlinger, 2013; Gerken et al., 2015).

Both developmental and adult acclimations, when applied alone, increased cold tolerance of *D. suzukii*. However, the promoting effect was higher when these two acclimation forms were combined. Developmental and adult acclimations are well known to increase cold tolerance of *D. melanogaster* (Sinclair and Roberts, 2005; Rako and Hoffmann, 2006; Overgaard et al., 2008; Koštál et al., 2011a; Colinet and Hoffmann, 2012) and *D. suzukii* (Jakobs et al., 2015). Here, we found that adult acclimation improved both survival and Ct_min_ more intensely than developmental acclimation, though these two acclimation treatments resulted in similar CCRT. It is assumed that the thermal conditions experienced just before a stress mainly determine subsequent cold tolerance (Geister and Fischer, 2007; Fischer et al., 2010; Colinet and Hoffmann, 2012). Thus, the higher effect of adult acclimation on cold tolerance could be due to its “temporal proximity” with the acute cold stress, whereas seven days at 25°C separated developmental acclimation from the cold shock (Figure 1). It is also possible that flies from developmental acclimation deacclimated during this period.

In this study, light cycles were not strictly identical among the experimental treatments. However, we reasoned that temperature is the main driver of the observed phenotypic changes (cold tolerance). Bauerfeind et al. (2014) reported that stress resistance traits (cold, heat, starvation, and desiccation resistance) are predominantly affected by temperature and not by photoperiod in *D. melanogaster*. Likewise, in *D. suzukii*, delayed reproductive maturity (i.e., the reproductive diapause syndrome) seems temperature-dependent and not regulated by photoperiod (Toxopeus et al., 2016). We therefore assumed that effects observed here on cold tolerance and physiology were primarily due to temperature.

### Acclimation Triggers Metabolic Changes in *D. suzukii*

One aim of this study was to identify metabolic changes resulting from different forms of cold acclimation in *D. suzukii*. We expected that the most cold-tolerant phenotypes would be characterized by accumulation of cryoprotectant molecules, such as carbohydrates, polyols or amino acids. GC-MS analysis revealed four non-overlapping metabotypes, suggesting different metabolic profiles among the four phenotypic groups (Figure 4). Amounts of several carbohydrates, such as Fru, Mal, Man, Suc, and Tre, increased in flies submitted to combined acclimation compare to controls (Supplementary Figure S4). Carbohydrates are known to be involved in cold tolerance of several insect species (Baust and Edwards, 1979; Kimura, 1982; Fields et al., 1998; Zeng et al., 2008; Ditrich and Koštál, 2011). In *D. melanogaster*, cold acclimation and rapid cold hardening increase the level of Fru, Glc, Mal, Suc, and Tre (Overgaard et al., 2007; Koštál et al., 2011a; Colinet et al., 2012). Even if caution should be exercised in designating a function to any upregulated or downregulated metabolite, as flux and pathways are unknown, the increased sugar level after combined acclimation in *D. suzukii* reverberates the results of Shearer et al. (2016). These authors found upregulations of gene clusters involved in the carbohydrates’ metabolism in winter morphs of *D. suzukii*. In this species, the cold-hardy “winter morph” is generated using a combination of developmental and adult acclimation (Stephens et al., 2015; Shearer et al., 2016; Toxopeus et al., 2016; Wallingford and Loeb, 2016). Unlike proper cold tolerant overwintering species that accumulate several hundred mmol L^-1^ of cryoprotectants (Salt, 1961), carbohydrate accumulation in cold acclimated drosophilids is of rather low magnitude, suggesting a non-colligatively contribution to cold hardiness (Koštál et al., 2011a; Colinet et al., 2012), for instance by stabilizing macromolecule structures (Arakawa and Timasheff, 1982; Crowe et al., 1988; Cacela and Hincha, 2006).

In comparison to controls, flies from the adult acclimation had increased concentrations of several polyols (glycerol, mannitol, and xylitol; Supplementary Figure S3). However, as for sugars, the concentrations and magnitude of changes remained too low to consider this pattern as a proper cryoprotective and colligative response. Polyols, and especially glycerol, are common cryoprotectants in cold-adapted insects (Sømme, 1982; Storey and Storey, 2005; Denlinger and Lee, 2010). Accumulation of massive concentrations of polyol are linked to freeze-protective functions such as the diminution of the supercooling point (Crosthwaite et al., 2011). On the other hand, at low concentrations, polyols may play other protective functions, likely through a “preferential” exclusion of solutes from proteins, which help to stabilize their structures (Gekko and Timasheff, 1981; Koštál et al., 2011a). In drosophilids, however, there is no clear evidence for substantial polyols accumulation correlated with cold hardiness (Kimura, 1982; Kelty and Lee, 2001; Overgaard et al., 2007; Koštál et al., 2011a; Colinet et al., 2012).

Flies subjected to adult acclimation showed a low level of spermine (Supplementary Figure S2). Polyamines are involved in stress tolerance in plants (Gill and Tuteja, 2010) and putatively in insects (Michaud et al., 2008). Previous reports have detected increase in some polyamines (putrescine and cadaverine) in cold acclimated flies (Koštál et al., 2011a; Colinet et al., 2012). However, there is no report on variations of spermine levels in response to acclimation. Spermine is a polyamine formed from spermidine, and it has been shown to mediate stress resistance in *Drosophila* (Minois et al., 2012). It remains unclear why spermine was specifically at low levels in adult acclimated flies. Polyamine metabolism is very dynamic and low level of spermine may be due to synthesis of spermidine. Unfortunately, we could not detect this latter metabolite.

Flies from combined acclimation were also characterized by accumulation of several amino acids (Ala, Ile, Leu, Phe, Ser, and Val; Supplementary Figure S1) in comparison to control. Amino acids are known to possess cryoprotective properties. For example, Pro is responsible of increasing cold and freeze tolerance in *D. melanogaster* (Koštál et al., 2012), and it allows larvae from the fly *Chymomyza costata* to survive exposure to liquid nitrogen (Koštál et al., 2011c). Here, the concentration of Pro did not change dramatically in response to combined acclimation, while it increased in response to acclimation in *D. melanogaster* (Koštál et al., 2011a). The increased concentrations of the other amino acids were relatively small compared to changes observed with Pro in *C. costata* by Koštál et al. (2011c). In addition to Pro, many other free amino acids (mainly Arg, but also Ile, Leu, Val, and Ala) can promote flies’ cold tolerance when supplemented in food (Koštál et al., 2016a). Although with different protocols, cold acclimation in *D. melanogaster* also triggered the accumulation of various amino acids in both adults (e.g., Val, Leu, Ser, Thr, Ile) (Colinet et al., 2012) and larvae (e.g., Pro, Asn, His, Glu) (Koštál et al., 2011a). In other insect species, cold acclimation or rapid cold hardening has consistently been correlated with the accumulation of various amino acids, among which Ala was often represented (Morgan and Chippendale, 1983; Storey, 1983; Hanzal and Jegorov, 1991; Fields et al., 1998; Michaud and Denlinger, 2007; Li et al., 2015). Here, Ala was the only amino acid showing the highest concentration after adult or combined acclimation (Supplementary Figure S1). The functional reasons for these amino acids accumulations in cold-resistant phenotypes are not clearly understood, but it might relate to metabolic protection or stabilization of macromolecules (Yancey, 2005). Cold-protective mechanisms of free amino acids (against freezing mainly) have been extensively discussed by Koštál et al. (2016a) who suggested a range of different interactions, such as preferential exclusion, protection of native protein structure, binding of partially unfolded proteins, stabilization of membrane structure and vitrification. Apart from these cold-protective functions, many amino acids are components of biosynthetic pathways linked to glycolysis and TCA cycle; therefore, these changes may also reflect consequences of perturbations of the metabolic rate that often characterize acclimation (Lu et al., 2014; Li et al., 2015). Similarly, Koštál et al. (2011b) observed a moderate accumulation of amino acids, such as Pro, Gln, and Ala, in overwintering *Pyrrhocoris apterus*, which probably resulted from a decrease of TCA cycle turnover rate, due to metabolic alteration induced by cold temperatures.

Here, we found that flies submitted to adult and combined acclimation were characterized by higher amounts of TCA intermediates than control flies or flies acclimated during development only (Figure 3). This could be linked to changes in metabolic rate. Indeed, metabolic cold adaptation or temperature compensation theories presume that at a same temperature, cold hardy insects showed similar or even higher metabolic rate than cold sensitive individuals (Hazel and Prosser, 1974; Chown and Gaston, 1999). Even if these theories are not always supported, several studies showed that cold acclimated insects had higher metabolic rate than warm acclimated ones (Terblanche et al., 2005; Isobe et al., 2013) and this may explain, at least in part, the changes in levels of TCA intermediates.

Despite showing unique metabotype, flies from developmental acclimation did not show drastic changes in their metabolite composition as observed in flies from combined and adult acclimation. Cold acclimation is a reversible trait (Slotsbo et al., 2016), therefore the moderate difference between flies from developmental acclimation and controls may be due to deacclimation. Despite these light metabotype divergences, our data showed that flies from developmental acclimation were more cold tolerant than control, based on all tested proxies. This suggests that physiological changes occurring during development, not only metabolic adjustments, persists and even carried over in adult stage, even if temperature returns permissive at adult stage.

### Temporal Trajectories of Metabotypes After Acute Cold Stress

Maintenance of metabolic homeostasis has been repeatedly associated with thermal tolerance in *D. melanogaster* (Malmendal et al., 2006; Colinet et al., 2012; Williams et al., 2014), although the functional mechanisms behind this correlated pattern are unknown and likely highly complex. We thus expected signs of metabolic inertia in cold acclimated phenotypes and signs of metabolic deregulation in chill-susceptible flies. Temporal analysis of metabotypes revealed different temporal patterns and mirrored cold tolerance of the four phenotypes (Figure 6). Control flies (the least cold tolerant) described a large and persistent metabolic deviation from initial state, suggesting a loss of metabolic homeostasis. Conversely, the most cold tolerant flies (flies from combined acclimation) showed only light initial deviation of metabolic trajectories followed by a rapid return to initial state during recovery period, suggesting a metabolic robustness and an efficient homeostatic response. Flies submitted to developmental and adult acclimation had intermediate cold tolerance and showed intermediate temporal metabolic response: initial deviation followed by incomplete return to initial metabolic state. Loss of homeostasis is likely related to development of thermal injuries (Malmendal et al., 2006) and several studies reported similar chill-induced homeostatic disruption in insects (Overgaard et al., 2007; Colinet et al., 2012; Teets et al., 2012; Williams et al., 2014).

Effects of chilling injuries can be immediate, accumulative or latently expressed (e.g., delayed mortality) (Turnock and Bodnaryk, 1991; Leopold, 2000; Overgaard et al., 2005). We noted that mortality after acute cold stress increased gradually with time of observation in control and flies acclimated during development suggesting latent damages. In contrast, flies that have been subjected to adult acclimation, combined or not with developmental acclimation, showed high and constant survival rates. Temporal increase of chill injuries in control and flies acclimated during development may be correlated with the temporal metabolic disorders that presented the highest distortion amplitudes in these two phenotypes.

The first axis of PCA, which described the temporal deviation of metabolic profiles, was correlated with accumulation of several amino acids, including several essential amino acids (Ile, Leu, Phe, Thr, and Val, Figure 6). Furthermore, the most chill-susceptible flies (control and developmental acclimation) were characterized by a temporal augmentation of the global amino acids pool (Figure 5). Such accumulation of amino acids after a cold stress has been reported in several insect species and is assumed to relate to protein breakdown, especially for essential amino acids, which can’t be synthetized *de novo* (Lalouette et al., 2007; Overgaard et al., 2007; Koštál et al., 2011b; Colinet et al., 2012). However, direct evidence of cold-induced protein degradation is scarce (Hochachka and Somero, 2002).

Chill-susceptible phenotypes (mainly control flies and to a lesser extent flies from developmental acclimation) were characterized by temporal accumulation of several polyols (e.g., erythritol, sorbitol, and inositol) after the acute cold stress (Supplementary Figure S10). As discussed previously, accumulation of polyols after acclimation or rapid cold hardening may have protective functions (Walters et al., 2009; Crosthwaite et al., 2011). Increase in polyol concentrations after a cold stress is also a general response in insects (Yoder et al., 2006; Lalouette et al., 2007; Michaud et al., 2008; Colinet et al., 2012). So far, it is not clear whether these accumulations have protective values or whether they represent biomarkers of complex metabolic deregulations due to cold stress (Colinet et al., 2012). Since only the two least cold-tolerant phenotypes (flies from control and developmental acclimation) displayed post-stress temporal increase in the level of some polyols, we can speculate that this most likely reflects a degenerative rather than a protective syndrome.

Concentration of amino acid pool didn’t change in time after the cold stress in flies from combined acclimation (Figure 5), suggesting low magnitude of cold injuries to proteins structures. Conversely, global carbohydrates and TCA intermediates pools decreased with time in these flies (Figure 5). This may relate to rapid mobilization of energetic metabolism during recovery. Williams et al. (2016) reported that *D. melanogaster* lines selected for cold tolerance presented metabolic rate depression during cold stress, but that metabolic rate increased in a higher proportion than in chill-susceptible flies during recovery from a cold exposure at 0°C. In the beetle *Alphitobius diaperinus*, recovery from cold stress during thermal fluctuation is also associated with an important metabolic rate augmentation (Lalouette et al., 2011). Repairing chilling injuries and re-establishing homeostasis likely induces energetic cost (MacMillan et al., 2012); therefore, reduction of TCA intermediates in flies from combined acclimation may be related to energy requirement for recovery.

## Conclusion

This study revealed that combining both developmental and adult cold acclimation resulted in a particularly high expression of cold tolerance in *D. suzukii*. This fly is believed to overwinter on wooded areas under leaf litters (Kanzawa, 1936), and trappings in autumn and winter revealed that captured flies were bigger, darker and more cold tolerant than flies captured in summer (Shearer et al., 2016). During these periods, flies are maybe developing slowly in infected fruits (protected in the litter) and then cold-acclimate as adults. Emerging flies by the end of winter season may therefore express high level of cold survival (at temperatures as low as -5°C) in the field. Our results indicate that cold tolerance plasticity of *D. suzukii* relies on physiological strategies similar to other drosophilids. Indeed, as frequently found in others drosophilids (e.g., Koštál et al., 2011a; Colinet and Hoffmann, 2012), we found that cold-acclimated *D. suzukii* accumulated low levels of cryoprotectants, such as sugars and amino acids, and were able to maintain metabolic homeostasis following cold stress. We highlighted that different acclimation treatments resulted in clearly distinct metabotypes, suggesting that physiological responses highly depend on thermal history. Collectively, these data contribute to the emerging understanding of the physiological strategies used by *D. suzukii* to acquire cold tolerance. The present metabolic analyses provided correlative but not causative effects of cold acclimation. Artificial variations of the candidate metabolites, for instance by diet supplementation or by disrupting their biosynthesis, could shed light on the function(s) of these metabolites in cold tolerance acquisition. In addition to metabolic adjustments and mobilization of cryoprotectants, cold tolerance plasticity relies on many other mechanisms and pathways, such as phospholipidic remodeling, maintenance of contractile function or altered thermal sensitivity of ion channel kinetics (Overgaard and MacMillan, 2016). Future studies should also explore these processes to gain better understanding of cold adaption of *D. suzukii*.

## Data Availability

The raw data supporting the conclusions of this manuscript will be made available by the authors on request, without undue reservation, to any qualified researcher.

## Author Contributions

TE and HC designed the experimental plan. TE conducted all experiments under the supervision of HC, DR, and MC. TE and HC analyzed the data and performed statistical analysis. TE and HC drafted the manuscript. All authors reviewed the manuscript.

## Conflict of Interest Statement

The authors declare that the research was conducted in the absence of any commercial or financial relationships that could be construed as a potential conflict of interest.
