# Supplementary material for: Cold Acclimation Favors Metabolic Stability in Drosophila suzukii
Source: Front Physiol. 2018 Nov 1;9:1506. doi: 10.3389/fphys.2018.01506 (PMC6221910; doi:10.3389/fphys.2018.01506)
Supplement: Supplementary file 1 [file Data_Sheet_1.pdf]

Supplementary Material

Cold acclimation favors metabolic stability in *Drosophila suzukii*

Thomas ENRIQUEZ[1], David RENAULT[1,2], Maryvonne CHARRIER[1] and Hervé COLINET[1*]

[1] ECOBIO - UMR 6553, Univ Rennes 1, CNRS, Rennes, France

[2] Institut Universitaire de France, 1 Rue Descartes, 75231 Paris cedex 05, France

***Correspondence :**

Hervé Colinet, Université de Rennes, CNRS, ECOBIO - UMR 6553, 263 Avenue du Général Leclerc, 35042 Rennes, France. Tel: +33 (0)2 23 23 64 38; email: herve.colinet@univ-rennes1.fr

Supplementary Figures

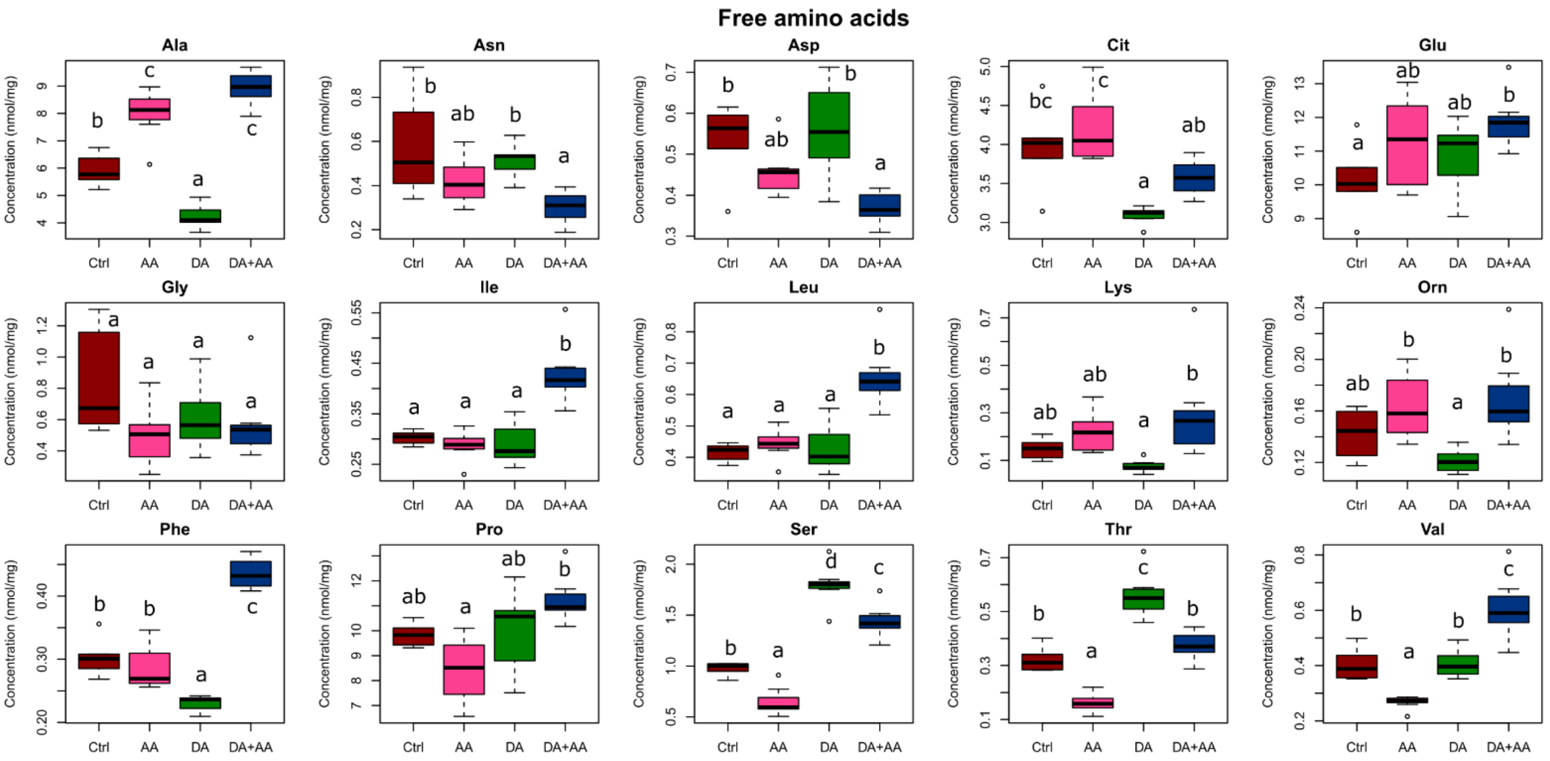

Figure S1: Concentrations of each free amino acid after the acclimation period. Boxplot sharing a same letter are not significantly different (*p*.value < 0.05; Tukey test). Ctrl: Control flies; DA: developmental acclimation; AA: adult acclimation; DA+AA: combined acclimation.

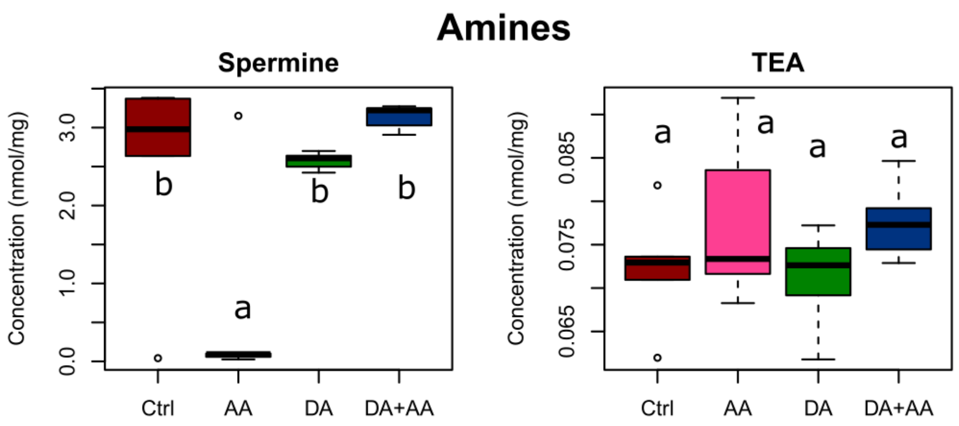

Figure S2: Concentrations of each amine after the acclimation period. Boxplot sharing a same letter are not significantly different (*p*.value < 0.05; Tukey test). Ctrl: Control flies; DA: developmental acclimation; AA: adult acclimation; DA+AA: combined acclimation.

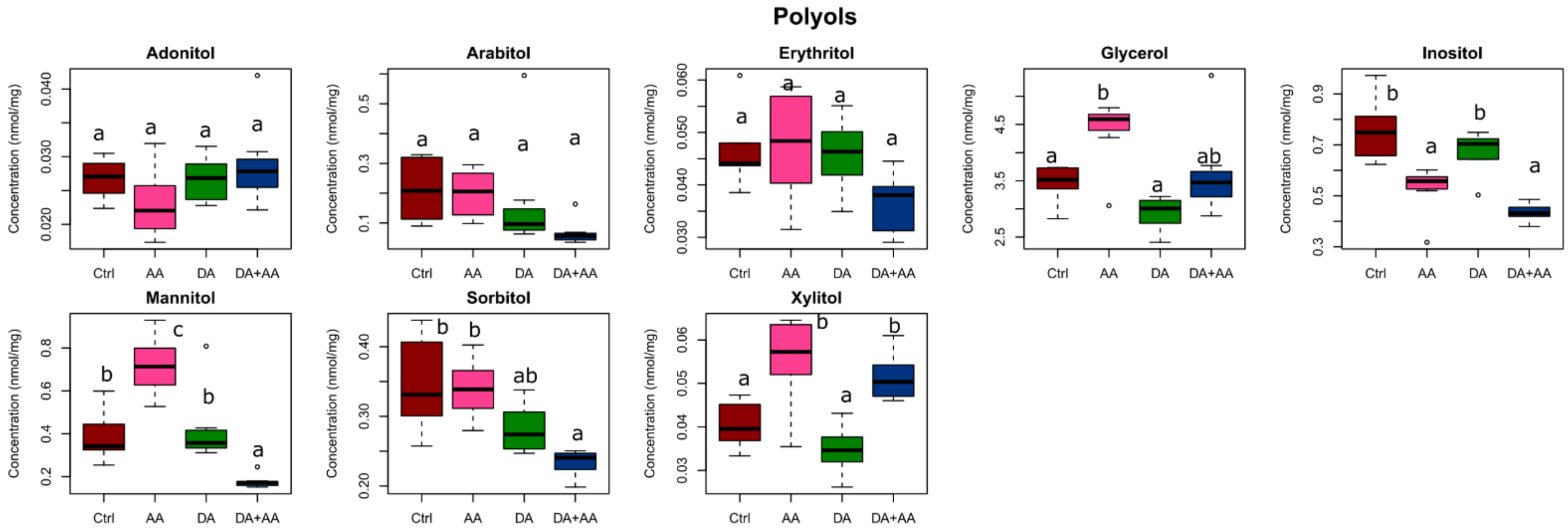

Figure S3: Concentrations of each polyol after the acclimation period. Boxplot sharing a same letter are not significantly different (*p*.value < 0.05; Tukey test). Ctrl: Control flies; DA: developmental acclimation; AA: adult acclimation; DA+AA: combined acclimation.

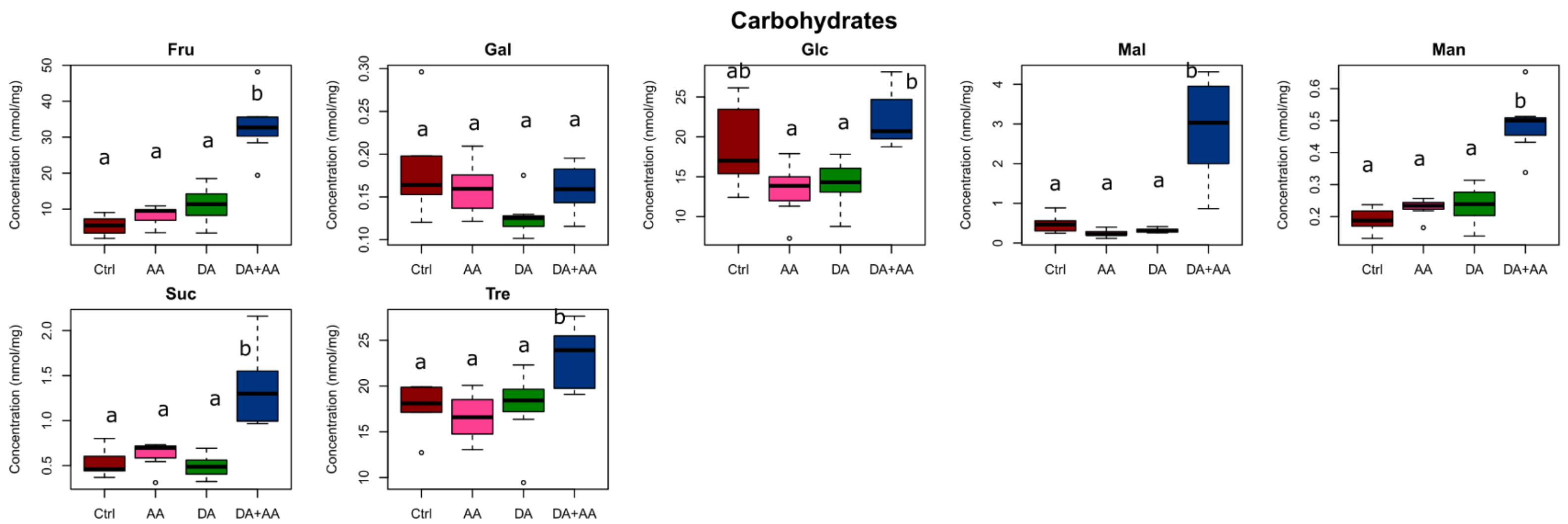

Figure S4: Concentrations of each carbohydrate after the acclimation period. Boxplot sharing a same letter are not significantly different (*p*.value < 0.05; Tukey test). Ctrl: Control flies; DA: developmental acclimation; AA: adult acclimation; DA+AA: combined acclimation.

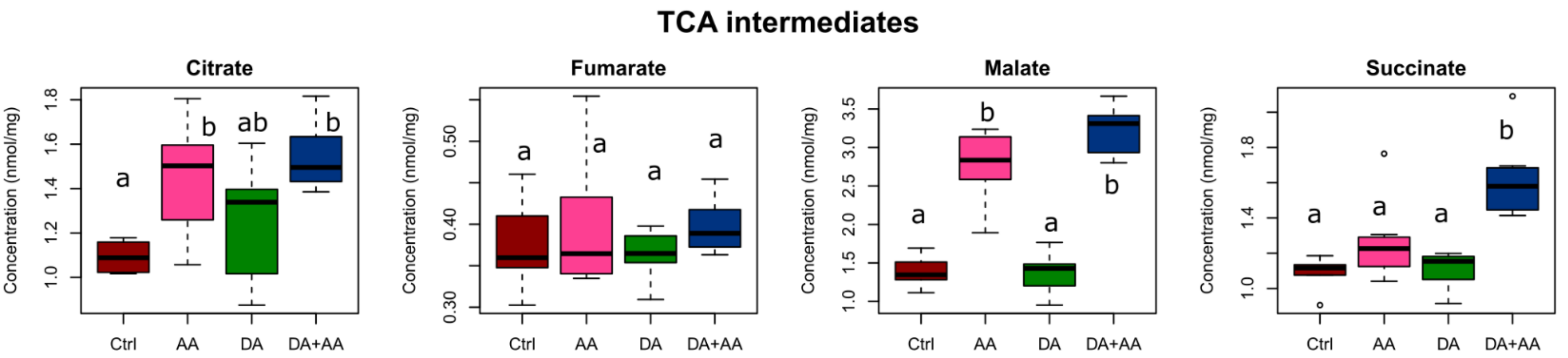

Figure S5: Concentrations of each TCA intermediate after the acclimation period. Boxplot sharing a same letter are not significantly different (*p*.value < 0.05; Tukey test). Ctrl: Control flies; DA: developmental acclimation; AA: adult acclimation; DA+AA: combined acclimation.

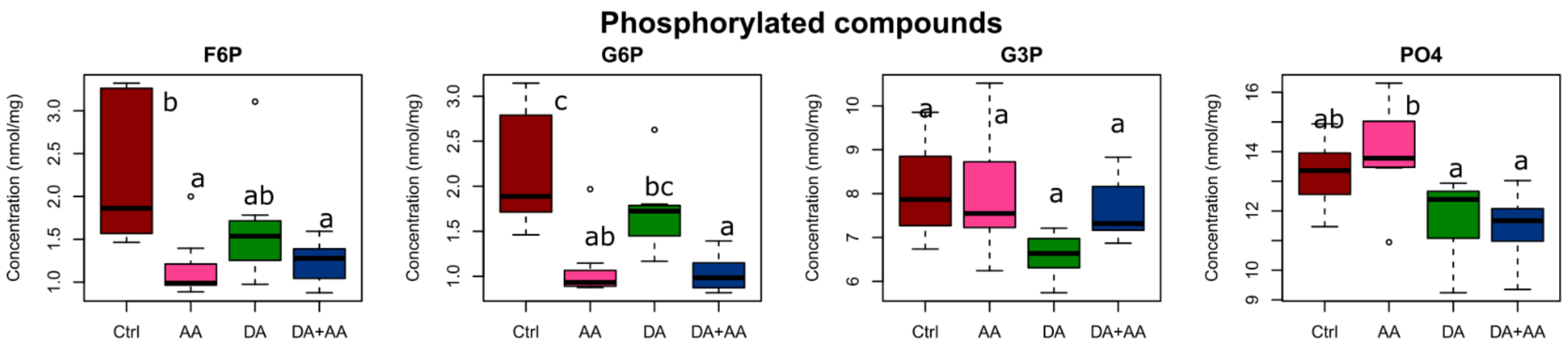

Figure S6: Concentrations of each phosphorylated compound after the acclimation period. Boxplot sharing a same letter are not significantly different (*p*.value < 0.05; Tukey test). Ctrl: Control flies; DA: developmental acclimation; AA: adult acclimation; DA+AA: combined acclimation.

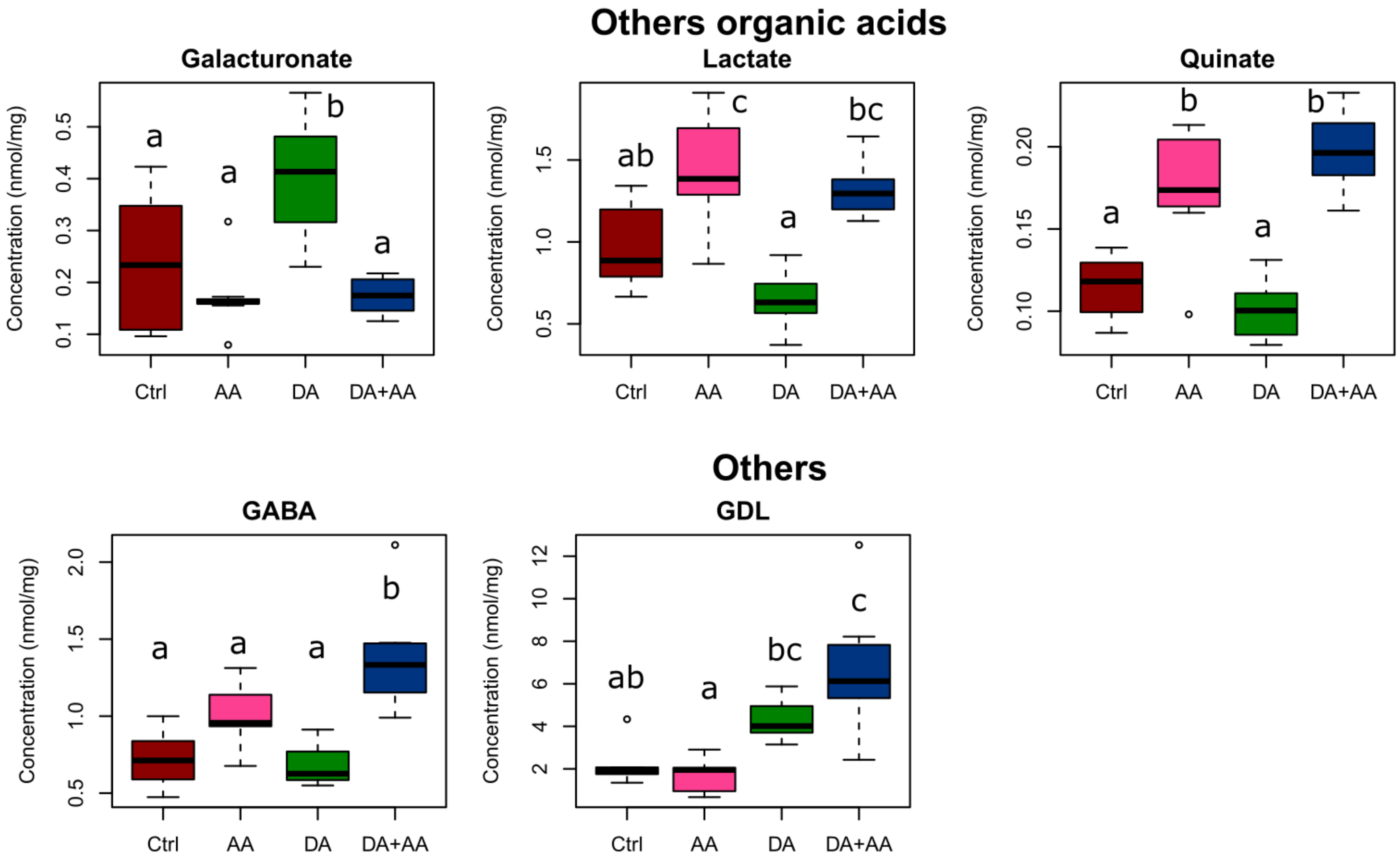

Figure S7: Concentrations of each other organic acids and other metabolites after the acclimation period. Boxplot sharing a same letter are not significantly different (*p*.value < 0.05; Tukey test). Ctrl: Control flies; DA: developmental acclimation; AA: adult acclimation; DA+AA: combined acclimation.

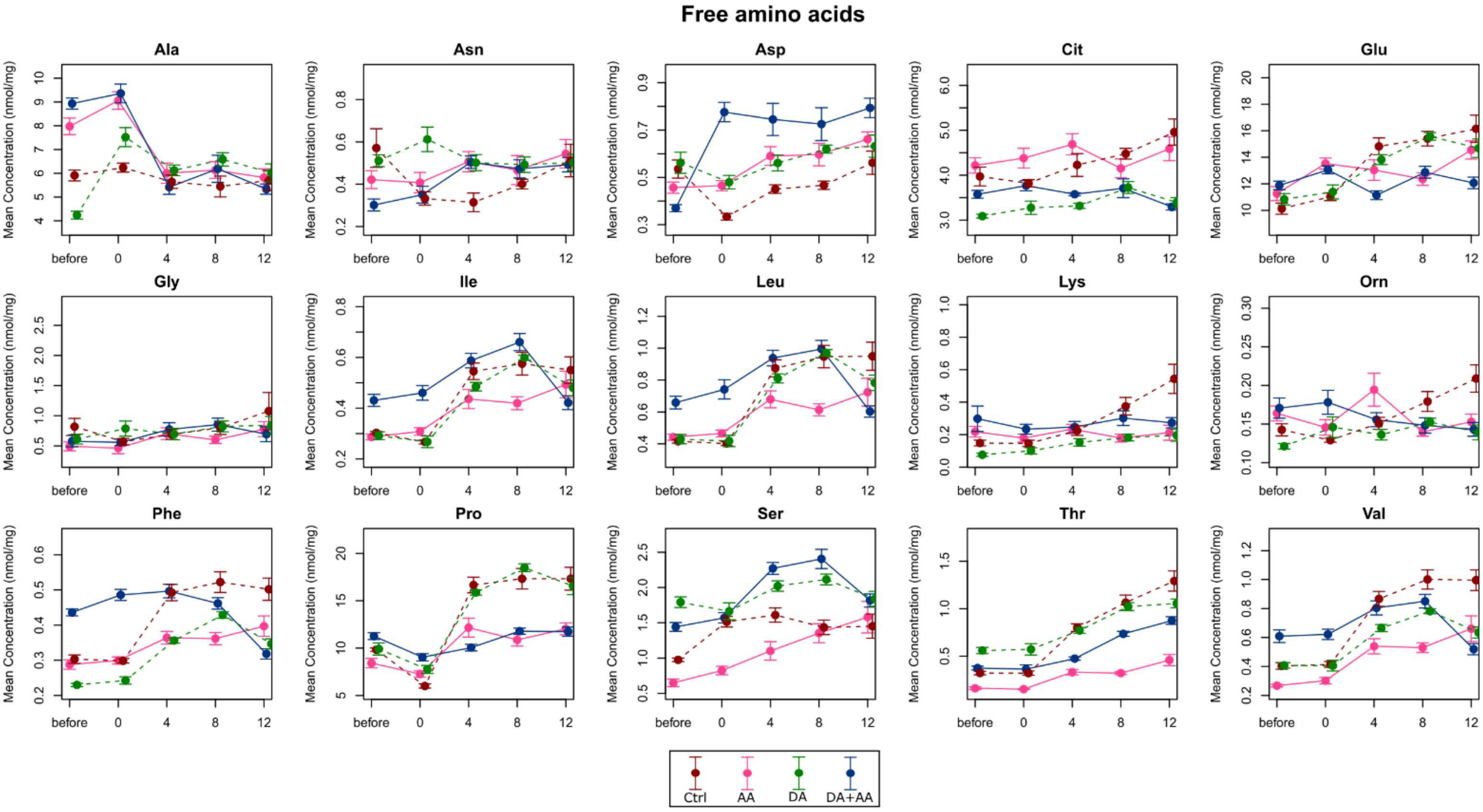

Figure S8: Temporal changes in concentrations of each free amino acid during the recovery from a cold stress at -5°C during 100 min (before, 0 h, 4 h, 8 h and 12 h after the cold stress). Ctrl: Control flies; DA: developmental acclimation; AA: adult acclimation; DA+AA: combined acclimation.

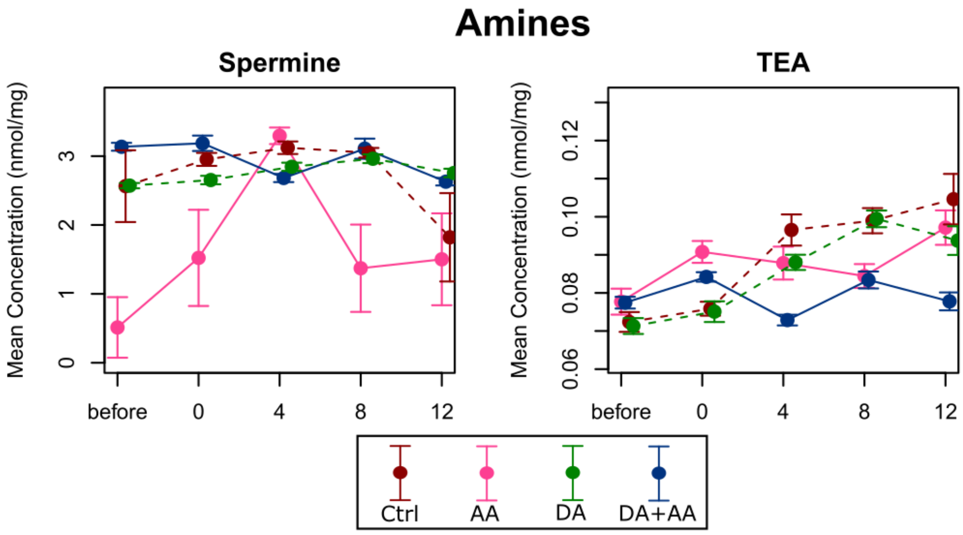

Figure S9: Temporal changes in concentrations of each amine during the recovery from a cold stress at -5°C during 100 min (before, 0 h, 4 h, 8 h and 12 h after the cold stress). Ctrl: Control flies; DA: developmental acclimation; AA: adult acclimation; DA+AA: combined acclimation.

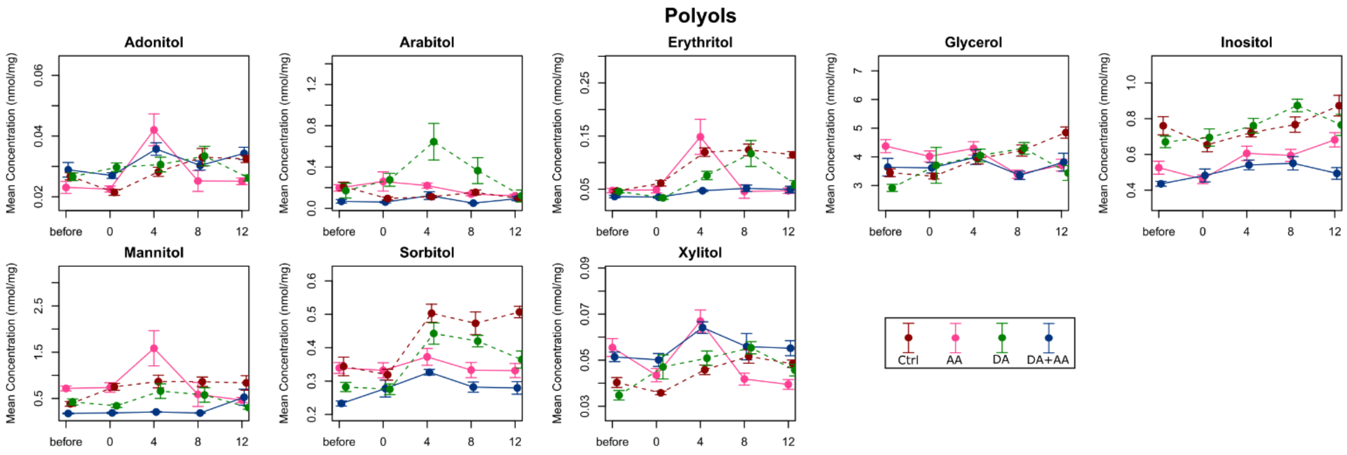

Figure S10: Temporal changes in concentrations of each polyol during the recovery from a cold stress at -5°C during 100 min (before, 0 h, 4 h, 8 h and 12 h after the cold stress). Ctrl: Control flies; DA: developmental acclimation; AA: adult acclimation; DA+AA: combined acclimation.

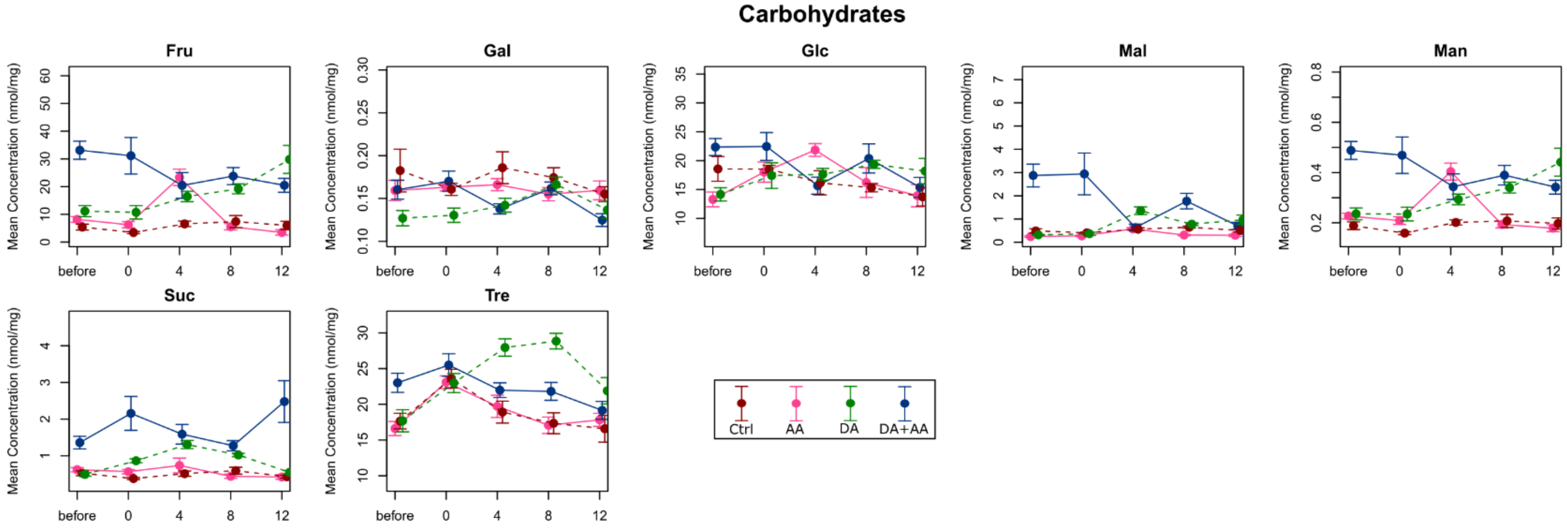

Figure S11: Temporal changes in concentrations of each carbohydrate during the recovery from a cold stress at -5°C during 100 min (before, 0 h, 4 h, 8 h and 12 h after the cold stress). Ctrl: Control flies; DA: developmental acclimation; AA: adult acclimation; DA+AA: combined acclimation.

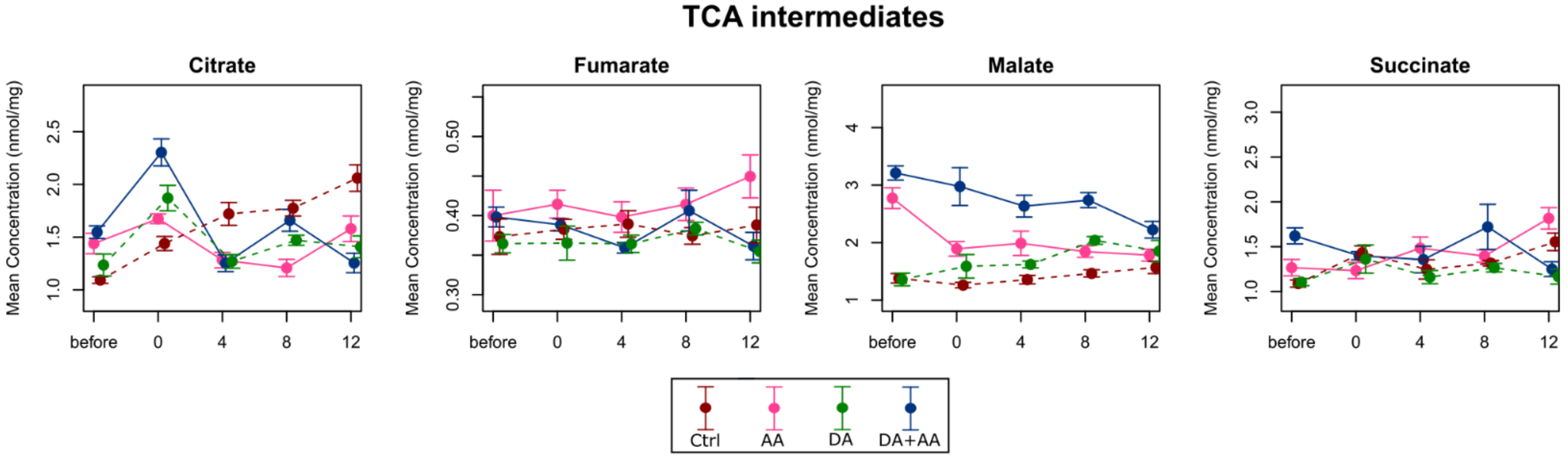

Figure S12: Temporal changes in concentrations of each TCA intermediate during the recovery from a cold stress at -5°C during 100 min (before, 0 h, 4 h, 8 h and 12 h after the cold stress). Ctrl: Control flies; DA: developmental acclimation; AA: adult acclimation; DA+AA: combined acclimation.

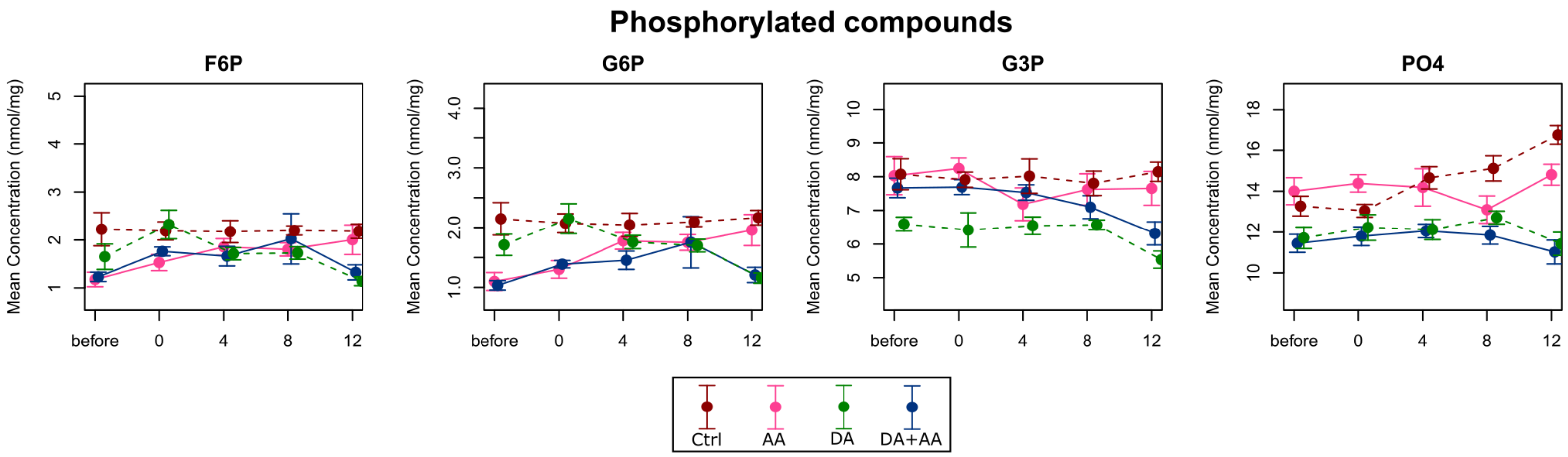

Figure S13: Temporal changes in concentrations of each phosphorylated compound during the recovery from a cold stress at -5°C during 100 min (before, 0 h, 4 h, 8 h and 12 h after the cold stress). Ctrl: Control flies; DA: developmental acclimation; AA: adult acclimation; DA+AA: combined acclimation.

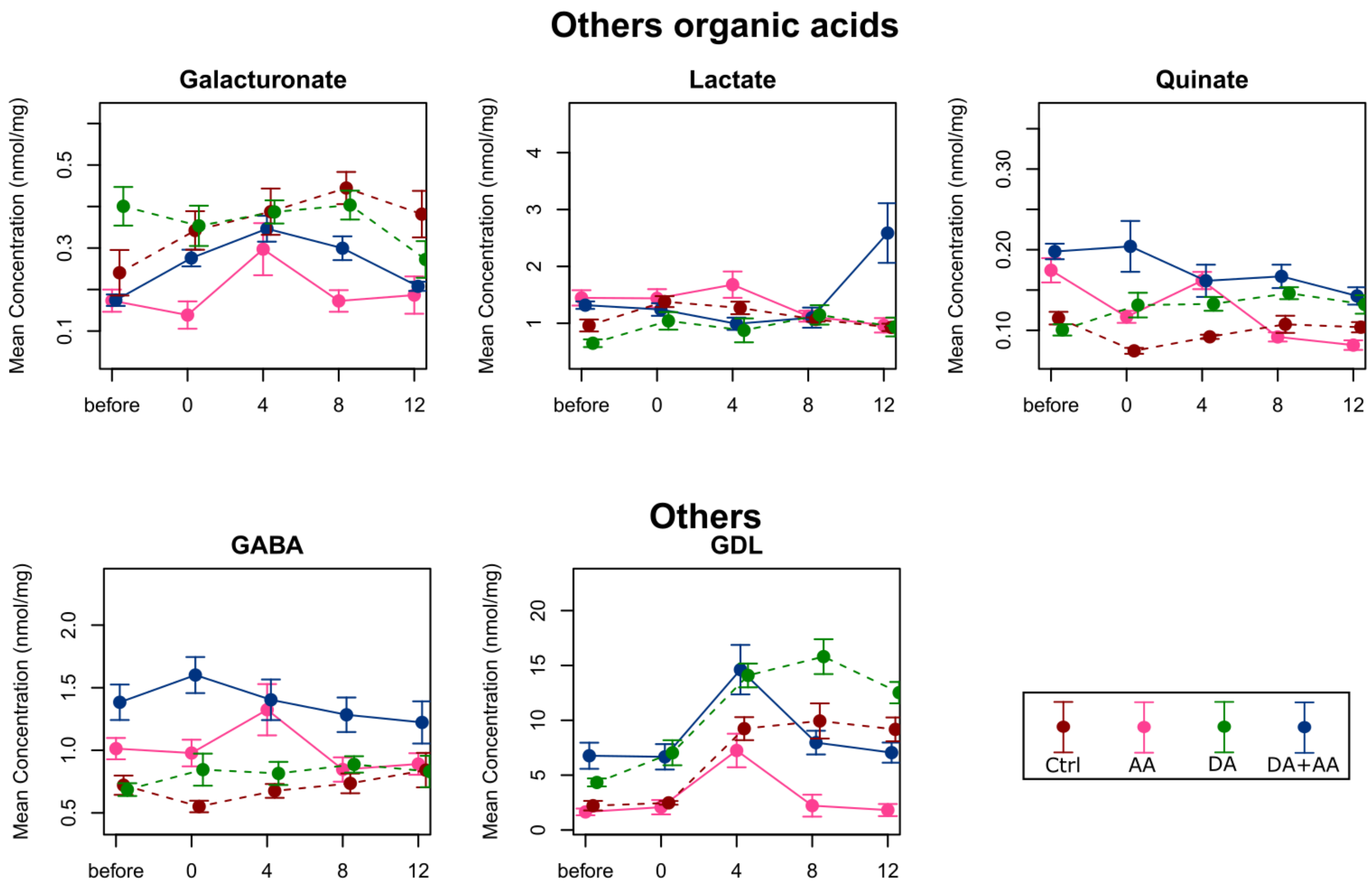

Figure S14: Temporal changes in concentrations of each other organic acids, and other metabolites during the recovery from a cold stress at -5°C during 100 min (before, 0 h, 4 h, 8 h and 12 h after the cold stress). Ctrl: Control flies; DA: developmental acclimation; AA: adult acclimation; DA+AA: combined acclimation.

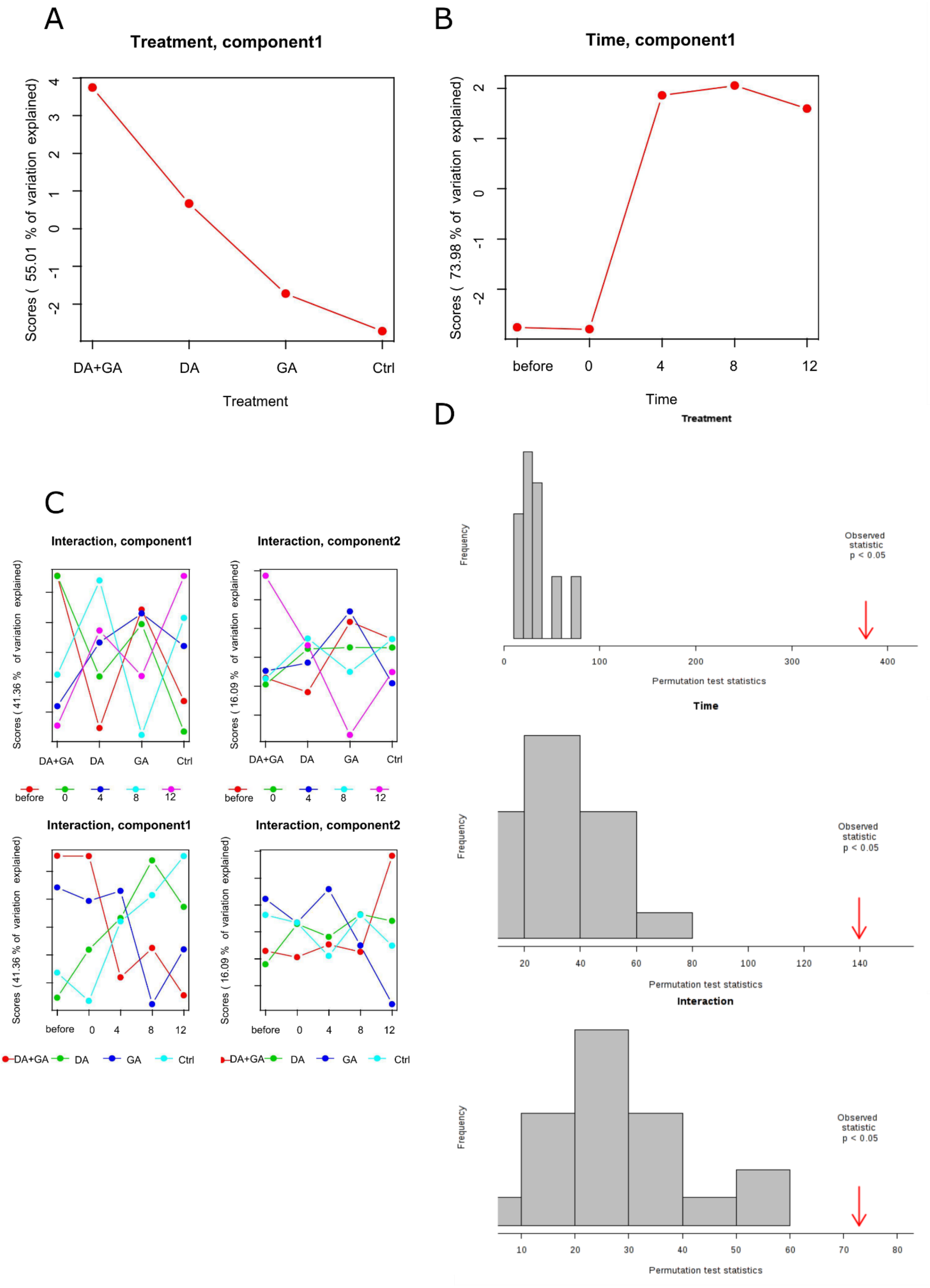
A
Treatment, component1
Scores (55.01 % of variation explained)
DA+GA
DA
GA
Ctrl
Treatment
B
Time, component1
Scores (73.98 % of variation explained)
before
0
4
8
12
Time
C
Interaction, component1
Scores (41.36 % of variation explained)
Interaction, component2
Scores (16.09 % of variation explained)
before
0
4
8
12
DA+GA
DA
GA
Ctrl
D
Treatment
Frequency
Observed statistic p < 0.05
Permutation test statistics
Time
Interaction

Figure S15: ANOVA - Simultaneous Component Analysis (ASCA), Metabolomic Data Analysis with MetaboAnalyst 4.0. ASCA is a multivariate extension of univariate ANOVA approach. It is designed to identify the major patterns associated with each factor. This implementation supports ASCA model for two factors with one interaction effect. The algorithm first partitions the overall data variance (X) into individual variances induced by each factor (A and B), as well as by the interactions (AB). The formula is shown below with (E) indicates the residual Errors: X = A + B + AB + E; A = Treatment (DA+AA, DA, AA, Ctrl); B = Time. The SCA part applies PCA to A, B, AB to summarize major variations in each partition. Users then detect the major pattern by visualizing the PCA scores plot. MetaboAnalyst also provides model validation to test the significance of the effects associated with main effects. It is based on the Manly's unrestricted permutation of observation then calculate the permuted variation associated with each factor. Major patterns associated with factor A (treatment) **(A)**. Major patterns associated with factor B (Time) **(B)**. Major patterns associated with interaction between the two factors **(C)**. Results of model validations through permutations **(D)**. Ctrl: Control flies; DA: developmental acclimation; AA: adult acclimation; DA+AA: combined acclimation.

Supplementary Tables

Table S1: Metabolites identified with GC-MS and their respective abbreviation. Metabolites are grouped by biochemical families.

**Free amino acids**	**Amines**	**Polyols**	**Carbohydrates**	**TCA intermediates**	**Phosphorylated compounds**	**Others organic acids**	**Others**
Alanine (Ala)	Spermine	Adonitol	Fructose (Fru)	Citrate	Fructose-6-phosphate (F6P)	Galacturonate	γ-Aminobutyric acid (GABA)
Asparagine (Asn)	Triethanolamine (TEA)	Arabitol	Galactose (Gal)	Fumarate	Glucose-6-phosphate (G6P)	Lactate	Gluconolactone (GDL)
Aspartate (Asp)		Erythritol	Glucose (Glc)	Malate	Glycerol-3-phosphate (G3P)	Quinate	
Citrulline (Cit)		Glycerol	Maltose (Mal)	Succinate	Phosphoric acid (PO4)		
Glutamate (Glu)		Inositol	Mannose (Man)				
Glycine (Gly)		Mannitol	Saccharose (Suc)				
Isoleucine (Ile)		Sorbitol	Trehalose (Tre)				
Leucine (Leu)		Xylitol					
Lysine (Lys)							
Ornithine (Orn)							
Phenylalanine (Phe)							
Proline (Pro)							
Serine (Ser)							
Threonine (Thr)							
Valine (Val)							

Table S2: Outcomes of GLMs on metabolites concentration during the recovery from the acute cold stress. TRT: thermal treatment; Time: recovery time.

Metabolites	Parameter	χ^2	df	*p*.value	Metabolites	Interaction	χ^2	df	*p*.value
Ala	TRT	50.46	3	< 0.001***	*Sorbitol*	TRT	122.67	3	< 0.001***
	Time	125.86	4	< 0.001***		Time	83.08	4	< 0.001***
	TRT x Time	124.26	12	< 0.001***		TRT x Time	49.10	12	< 0.001***
Asn	TRT	15.02	3	< 0.01**	*Xylitol*	TRT	33.67	3	< 0.001***
	Time	7.23	4	0.12		Time	43.61	4	< 0.001***
	TRT x Time	37.98	12	< 0.001***		TRT x Time	63.80	12	< 0.001***
Asp	TRT	78.93	3	< 0.001***	*Fru*	TRT	150.85	3	< 0.001***
	Time	58.16	4	< 0.001***		Time	3.98	4	0.41
	TRT x Time	77.62	12	< 0.001***		TRT x Time	78.83	12	< 0.001***
Cit	TRT	137.82	3	< 0.001***	*Gal*	TRT	23.16	3	< 0.001***
	Time	11.14	4	< 0.05*		Time	7.84	4	0.10
	TRT x Time	35.78	12	< 0.001***		TRT x Time	20.50	12	0.06
Glu	TRT	17.69	3	< 0.001***	*Glc*	TRT	8.26	3	< 0.05*
	Time	100.73	4	< 0.001***		Time	10.44	4	< 0.05*
	TRT x Time	89.66	12	< 0.001***		TRT x Time	32.63	12	< 0.01**
Gly	TRT	6.48	3	0.09	*Mal*	TRT	93.93	3	< 0.001***
	Time	12.01	4	< 0.05*		Time	5.99	4	0.20
	TRT x Time	9.80	12	0.63		TRT x Time	83.06	12	< 0.001***
Ile	TRT	46.45	3	< 0.001***	*Man*	TRT	127.37	3	< 0.001***
	Time	218.88	4	< 0.001***		Time	3.78	4	0.44
	TRT x Time	59.85	12	< 0.001***		TRT x Time	79.26	12	< 0.001***
Leu	TRT	46.22	3	< 0.001***	*Suc*	TRT	137.91	3	< 0.001***
	Time	228.28	4	< 0.001***		Time	6.25	4	0.18
	TRT x Time	87.74	12	< 0.001***		TRT x Time	37.25	12	< 0.001***
Lys	TRT	42.65	3	< 0.001***	*Tre*	TRT	53.67	3	< 0.001***
	Time	31.95	4	< 0.001***		Time	42.00	4	< 0.001***
	TRT x Time	49.62	12	< 0.001***		TRT x Time	49.73	12	< 0.001***
Orn	TRT	14.39	3	< 0.01**	*Citrate*	TRT	17.80	3	< 0.001***
	Time	3.53	4	0.47		Time	69.42	4	< 0.001***
	TRT x Time	57.34	12	< 0.001***		TRT x Time	129.64	12	< 0.001***
Phe	TRT	174.66	3	< 0.001***	*Fumarate*	TRT	19.32	3	< 0.001***
	Time	168.43	4	< 0.001***		Time	1.85	4	0.76
	TRT x Time	184.37	12	< 0.001***		TRT x Time	10.91	12	0.54
Pro	TRT	124.42	3	< 0.001***	*Malate*	TRT	228.65	3	< 0.001***
	Time	397.53	4	< 0.001***		Time	12.44	4	< 0.05*
	TRT x Time	145.52	12	< 0.001***		TRT x Time	57.98	12	< 0.001***
Ser	TRT	193.53	3	< 0.001***	*Succinate*	TRT	18.17	3	< 0.001***
	Time	88.24	4	< 0.001***		Time	7.40	4	0.12
	TRT x Time	50.74	12	< 0.001***		TRT x Time	39.81	12	< 0.001***
Thr	TRT	420.14	3	< 0.001***	*F6P*	TRT	21.44	3	< 0.001***
	Time	514.02	4	< 0.001***		Time	9.76	4	< 0.05*
	TRT x Time	113.16	12	< 0.001***		TRT x Time	22.33	12	< 0.05*
Val	TRT	115.62	3	< 0.001***	*G6P*	TRT	42.82	3	< 0.001***
	Time	237.29	4	< 0.001***		Time	8.35	4	0.08
	TRT x Time	99.91	12	< 0.001***		TRT x Time	32.56	12	< 0.01**
Spermine	TRT	46.36	3	< 0.001***	*G3P*	TRT	59.65	3	< 0.001***
	Time	16.17	4	< 0.01**		Time	8.98	4	0.06
	TRT x Time	34.11	12	< 0.001***		TRT x Time	12.57	12	0.40
TEA	TRT	31.47	3	< 0.001***	*PO4*	TRT	111.82	3	< 0.001***
	Time	88.25	4	< 0.001***		Time	6.32	4	0.18
	TRT x Time	78.67	12	< 0.001***		TRT x Time	35.68	12	< 0.001***
Adonitol	TRT	7.59	3	0.06	*Galacturonate*	TRT	64.34	3	< 0.001***
	Time	41.32	4	< 0.001***		Time	20.49	4	< 0.001***
	TRT x Time	49.35	12	< 0.001***		TRT x Time	21.67	12	< 0.05*
Arabitol	TRT	41.25	3	< 0.001***	*Lactate*	TRT	25.04	3	< 0.001***
	Time	15.72	4	< 0.01**		Time	6.09	4	0.19
	TRT x Time	36.77	12	< 0.001***		TRT x Time	67.36	12	< 0.001***
Erythritol	TRT	51.51	3	< 0.001***	*Quinate*	TRT	96.52	3	< 0.001***
	Time	73.80	4	< 0.001***		Time	14.14	4	< 0.01**
	TRT x Time	72.69	12	< 0.001***		TRT x Time	61.61	12	< 0.001***
Glycerol	TRT	6.29	3	0.10	*GABA*	TRT	94.81	3	< 2e-16***
	Time	9.65	4	< 0.05*		Time	2.74	4	0.60
	TRT x Time	48.55	12	< 0.001***		TRT x Time	18.36	12	0.11
Inositol	TRT	163.43	3	< 0.001***	*GDL*	TRT	128.68	3	< 0.001***
	Time	36.58	4	< 0.001***		Time	121.20	4	< 0.001***
	TRT x Time	21.74	12	< 0.05*		TRT x Time	53.84	12	< 0.001***
Mannitol	TRT	52.89	3	< 0.001***					
	Time	19.17	4	< 0.001***					
	TRT x Time	39.39	12	< 0.001***					
